# Switching the hydrogenation selectivity of urea derivatives *via* subtly tuning the amount and type of additive in the catalyst system†

**DOI:** 10.1039/d3sc05674k

**Published:** 2023-12-23

**Authors:** Jun Zhu, Yongtao Wang, Jia Yao, Haoran Li

**Affiliations:** a Department of Chemistry, ZJU-NHU United R&D Center, Zhejiang University Hangzhou 310027 China lihr@zju.edu.cn; b State Key Laboratory of Chemical Engineering, College of Chemical and Biological Engineering, Zhejiang University Hangzhou 310027 China

## Abstract

Catalytic hydrogenation of urea derivatives is considered to be one of the most feasible methods for indirect reduction functionalization of CO_2_ and synthesis of valuable chemicals and fuels. Among value-added products, methylamines, formamides and methanol are highly attractive as important industrial raw materials. Herein, we report the highly selective catalytic hydrogenation of urea derivatives to *N*-monomethylamines for the first time. More importantly, two- and six-electron reduction products can be switched on/off by subtly tuning 0.5 mol% KO^*t*^Bu (2% to 1.5%): when the molar ratio of KO^*t*^Bu/(PPh_3_)_3_RuCl_2_ exceeds 2.0, it is favorable for the formation of two-electron reduction products (*N*-formamides), while when it is below 2.0, the two-electron reduction products are further hydrogenated to six-electron reduction products (*N*-monomethylamines and methanol). Furthermore, changing the type of additive can also regulate this interesting selectivity. Control experiments showed that this selectivity is achieved by regulating the acid-base environment of the reaction to control the fate of the common hemiaminal intermediate. A feasible mechanism is proposed based on mechanistic experiments and characterization. This method has the advantages of being simple, universal and highly efficient, and opens up a new synthesis strategy for the utilization of renewable carbon sources.

## Abstract 2

We have developed a triphos-Ru catalytic system that can switch the chemoselectivity in catalytic hydrogenation of urea derivatives (from *N*-formamides to *N*-monomethylamines and methanol) by changing only 0.5 mol% KO^*t*^Bu (2% to 1.5%).

## Graphical abstract

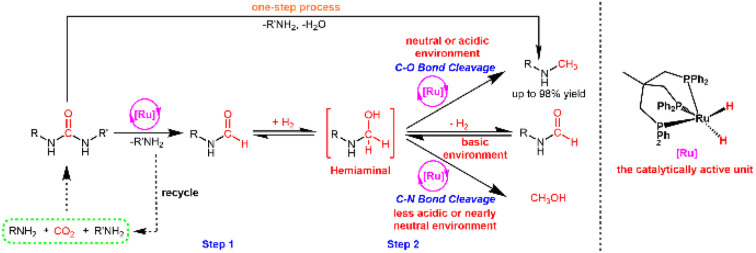

## Introduction

**Scheme 1:**
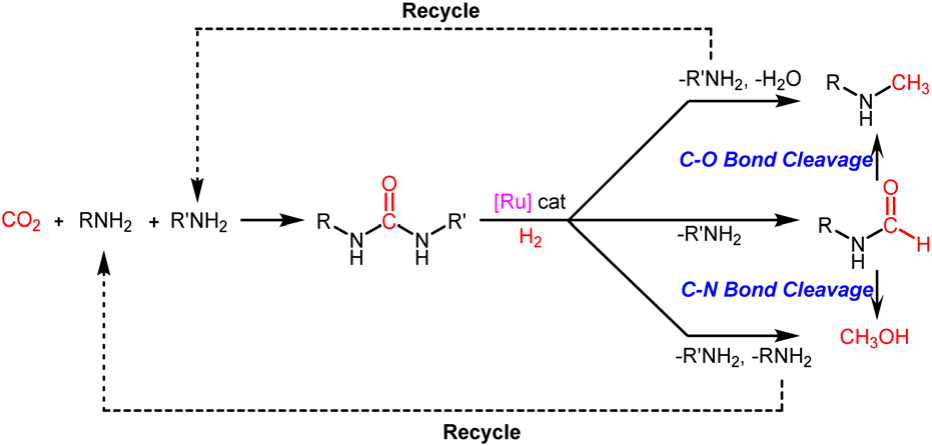
Indirect conversion of CO_2_ and amines to value-added chemicals *via* hydrogenation of urea derivatives.

In recent years, carbon dioxide (CO_2_) reduction and functionalization have provided new insights into its chemical conversion and utilization.^1–4^ However, due to thermodynamic and kinetic limitations, to date, only a few processes using CO_2_ as a C1 source have been industrialized and these have been mainly used for the production of urea and its derivatives.^5–8^ Against this backdrop, catalytic hydrogenation of urea derivatives to value-added chemicals is a promising way to expand the resource utilization of CO_2_ (Scheme 1).^9–13^

*N*-Methylamine compounds are important solvents and play an important role in platform chemicals such as medicine, perfumes, synthetic resins, agricultural chemicals and dyes.^14,15^ Traditionally, producing *N*-methylamines mainly requires formaldehyde or toxic methylation reagents such as methyl iodide, methyl triflate, dimethyl sulfate, or diazomethane.^16–19^ Notably, in the process of producing *N*-monomethylamines, a mixture of *N*-monomethylamine and *N*,*N*-dimethylamine is often obtained because the formation of *N*,*N*-dimethylamine is a thermodynamically favorable reaction.^20^ Thus, highly selective synthesis of *N*-monomethylamine is a challenge. In this perspective, highly selective catalytic hydrogenation of CO_2_-derived urea derivatives to methylamines and the corresponding recyclable amines is a novel and environmentally friendly approach. In addition, although direct hydrogenation of CO_2_ to methanol has been extensively studied, it involves relatively harsh reaction conditions.^21–26^ In industry, formamides are produced by the reaction of an amine with toxic and flammable CO in methanol using an NaOCH_3_ catalyst,^27–29^ and methylamines are obtained by the reaction of an amine with methanol,^30,31^ both of which require methanol as the raw material. Thus, hydrogenation of urea derivatives may be an alternative strategy for the synthesis of valuable chemicals and fuels from CO_2_ under mild reaction conditions, and the resulting amine byproducts can be recycled.

In 2011, Milstein and colleagues reported the first example for hydrogenation of urea derivatives to methanol.^10^ Subsequently, several catalytic systems, including Ru and Ir, have been used for the hydrogenation of CO_2_-derived urea derivatives to formamide products.^32–34^ Notably, formamides can be further hydrogenated to yield the six-electron reduction products, such as methylamines and methanol.^35–38^ At present, methanol is still the main 6-electron reduction product reported by hydrogenation of urea derivatives, and the research on obtaining methylamine compounds is still limited. To date, only Leitner has reported the formation of methylaniline (24% yield) from diphenylurea using Ru/triphos in the presence of acid additives, accompanied by the formation of dimethylamine (8%) and methanol byproducts.^32^ Inspired by this result, we attempted to achieve the highly selective hydrogenation of urea derivatives to the monomethylamine as the main product. More importantly, an example of good selectivity for all three types of products using a single well-defined catalyst system by adjusting the process conditions is even more lacking.

Obviously, this step from formamides to methylamines and methanol is the key to controlling the selective reduction of urea derivatives to two-electron (*N*-formamides) and six-electron reduction products (*N*-monomethylamines and methanol). In view of this, further controlled reduction of formamide products to methylamine and methanol products is a feasible path to expand the utilization of urea and its derivatives. In addition, with formamides it is difficult to achieve selectivity between deoxidation hydrogenation (C–O bond cleavage) and deammonia hydrogenation (C–N bond cleavage).^37,39,40^ In this context, it is of great significance to develop an efficient catalyst system for hydrogenation of urea derivatives, which can adjust the chemoselectivity to obtain two-electron (*N*-formamides) and six-electron reduction products (*N*-monomethylamines and methanol) with high selectivity.

Herein, in order to achieve hierarchical control of hydrogenated urea derivatives to two- and six-electron reduction products, we attempt to further reduce formamide products to *N*-monomethylamines and CH_3_OH by tuning the reaction parameters on the basis of a Ru-triphos catalyst system for semi-hydrogenation of urea derivatives to formamides.

## Results and discussion

In our previous study on the preparation of formamide from hydrogenated urea derivatives, we observed the formation of the 4-chloro-*N*-methylaniline by-product in addition to the *N*-(4-chlorophenyl)formamide main product using the Ru-triphos catalyst system in the absence of KO^*t*^Bu.^33^ However, no significant 4-chloro-*N*-methylaniline product was detected in the presence of 2.5 mol% KO^*t*^Bu. Based on these results, we wondered if further hydrogenation of formamide to 4-chloro-*N*-methylaniline could be facilitated by regulating the amount of KO^*t*^Bu used. It can be clearly seen in Table 1 that when the amount of KO^*t*^Bu added is 2 mol%, the 7% yield of *N*-methylamine (maximum yield is 100% based on the number of moles of urea, *i.e.*, the maximum total yields of amine and methylamine is 200%) can be observed (Table 1, entry 1). Lowering the KO^*t*^Bu loadings to 1.5 mol%, *N*-formamide was completely converted to *N*-methylamine (Table 1, entry 2). The results are surprising that just by changing 0.5 mol% KO^*t*^Bu (2% to 1.5%), the selectivity switches from 84% formamide to 80% *N*-methylated amine. As the KO^*t*^Bu loadings gradually decreased, the yield of methanol also decreased (entries 2–6). When the KO^*t*^Bu loadings decreased to as low as 0.125 mol%, the reaction rate decreased significantly and even the intermediate formamide could be detected (Table 1, entries 6–8). By comparison, it was found that the selectivity of *N*-monomethylamine was the highest in the presence of 0.25 mol% KO^*t*^Bu (Table 1, entry 5), but there was still a high content of 4-chloro-*N*,*N*-dimethylaniline product (8%). In order to improve the selectivity of *N*-monomethylamine, we continued to optimize the reaction conditions to reduce the amount of this byproduct. On the basis of the above optimization, the selectivity of *N*-monomethylamine was not improved with a catalyst loading of 2 mol% (Table 1, entry 9). Subsequently, we changed the reaction temperature and found that the yield of the *N*,*N*-dimethylamine by-product decreased with the increase of reaction temperature (Table 1, entries 10 and 11). When the reaction temperature was increased to 160 °C, only traces of *N*,*N*-dimethylamine could be detected, and the yield of 4-chloro-*N*-methylaniline was up to 98% (Table 1, entry 11). Interestingly, the selectivity of *N*-monomethylamine decreased when the hydrogen pressure was lowered, but the conversion rate did not decrease (Table 1, entries 12 and 13). Moreover, 89% and 81% yields of 4-chloro-*N*-methylaniline were achieved respectively when using NaHBEt_3_ or NaOH as additives instead of KO^*t*^Bu (Table 1, entries 14 and 15).

**Table 1:** Optimization of the catalytic conditions for hydrogenation of 1,3-bis(4-chlorophenyl)urea^a^

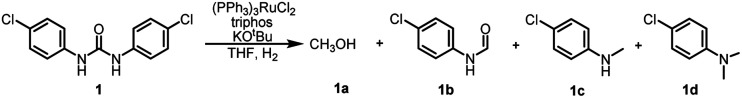								
Entry	KO^*t*^Bu (mol%)	H_2_ (bar)	Temperature (°C)	Time (h)	Yield (%) of 1a	Yield (%) of 1b	Yield (%) of 1c	Yield (%) of 1d
1	2	50	140	12	2	84	7	<1
2	1.5	50	140	12	9	0	80	6
3	1	50	140	12	7	0	79	8
4	0.5	50	140	12	2	0	83	7
5	0.25	50	140	12	1	0	87	8
6	0.125	50	140	12	<1	4	80	5
7	0.125	50	140	18	<1	0	85	6
8	0	50	140	24	<1	7	74	6
9^b^	0.25	50	140	8	2	0	81	8
10	0.25	50	150	10	1	0	96	2
11	0.25	50	160	8	<1	0	98 (93)	<1
12	0.25	30	160	8	5	0	91	1
13	0.25	10	160	8	19	0	73	1
14^c^	1	50	140	12	2	0	89	5
15^d^	1	50	140	12	3	0	81	7

a Reaction conditions: substrate (2 mmol), (PPh_3_)_3_RuCl_2_ (1 mol%), triphos (1.5 mol%), KO^*t*^Bu (0–2.5 mol%), H_2_ (10–50 bar), THF (4 mL), 140–160 °C (bath temperature). Determined by GC using biphenyl as an internal standard. Identification of the products was also confirmed by GC-MS and ^1^H NMR; isolated yield in parentheses.

b (PPh_3_)_3_RuCl_2_ (2 mol%) and triphos (3 mol%) were used.

c NaHBEt_3_ was used instead of KO^*t*^Bu.

d NaOH was used instead of KO^*t*^Bu.

After investigating the optimum reaction conditions, the substrate range of the ruthenium-catalyzed selective preparation of *N*-monomethylamines from urea derivatives was discussed (Table 2). We initially chose substrates with electron-withdrawing groups on the aniline ring, with yields of *N*-monomethylamines ranging from 79 to 98% (entries 2–5). For 1,3-diphenylurea without substituents on the aromatic ring, the yield of *N*-methylaniline was 98% using NaHBEt_3_ as an additive instead of KO^*t*^Bu. For 1,3-di(pyridin-2-yl)urea, which replaced the aromatic ring with pyridine, a moderate yield of 2-(methylamino)pyridine (61%) was obtained (entry 6). Surprisingly, substrates with strong electron-donating substituents on the aryl ring of aniline were also well tolerated, with 82–93% yields of the corresponding products (entries 7–9). As expected, asymmetric urea (1-(4-chlorophenyl)-3-(3,4-dichlorophenyl)urea) was also well converted to 4-chloro-*N*-methylaniline (65%) and 3,4-dichloro-*N*-methylaniline (22%) products (entry 10). The different yields may be related to the electronic and steric properties of substituents on the *N*-aromatic ring.

**Table 2:** Hydrogenation of urea derivatives to *N*-monomethylamines catalyzed by a triphos-based ruthenium catalyst system^a^

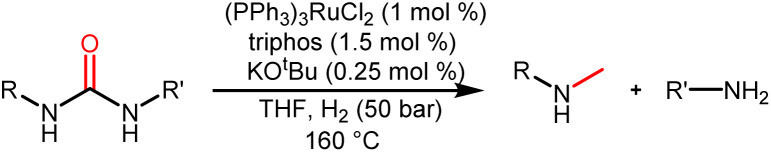					
Entry	Substrate	Time (h)	Product	Yield^b^ (%) of *N*-monomethylamine	Yield^b^ (%) of amine
1	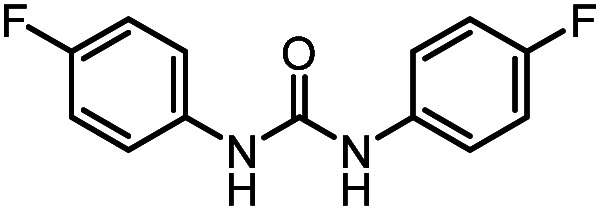	12	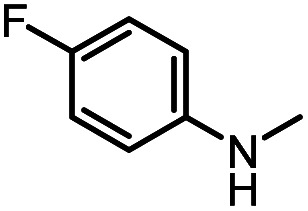	98 (94)	100
2	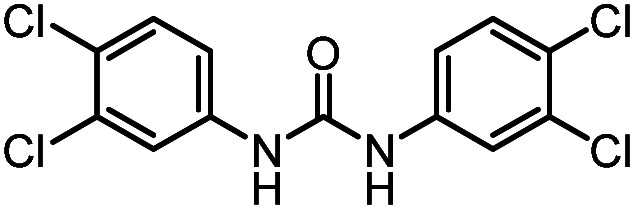	8	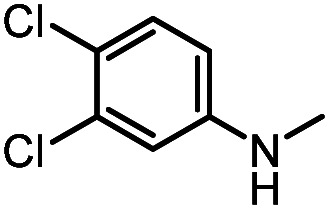	86	103
3	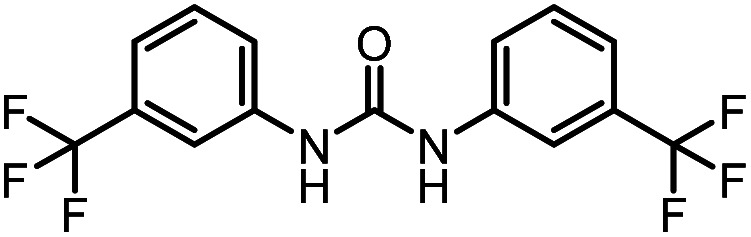	12	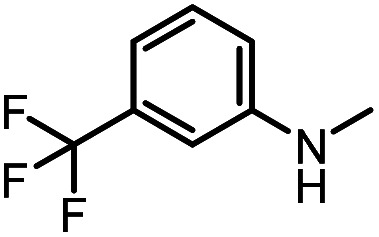	91	104
4	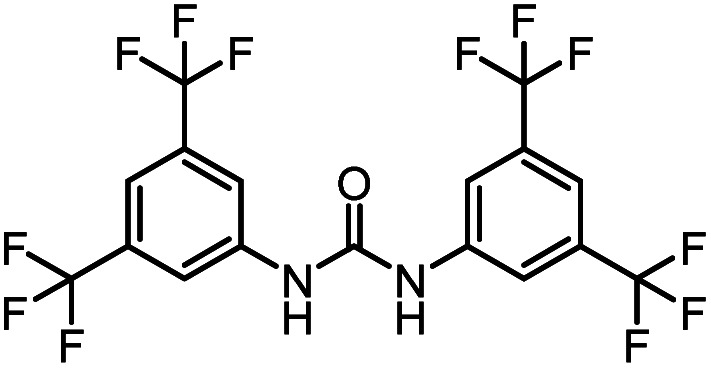	12	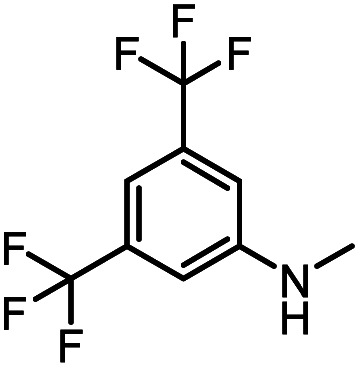	79	119
5	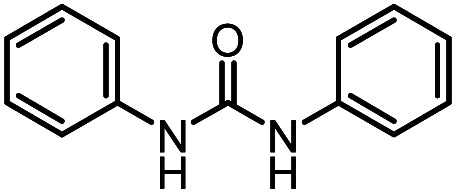	12^c^	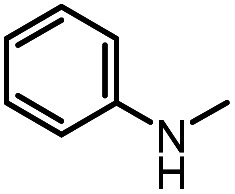	98 (91)	100
6	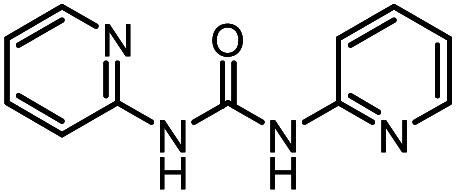	12	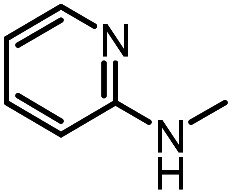	61	98
7	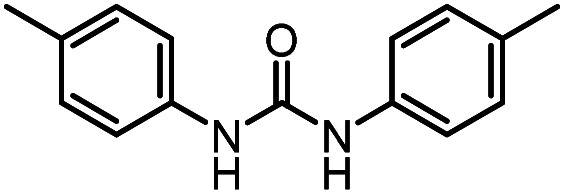	12	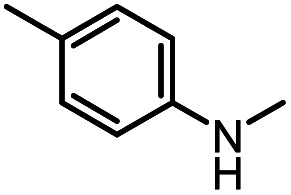	92 (89)	102
8	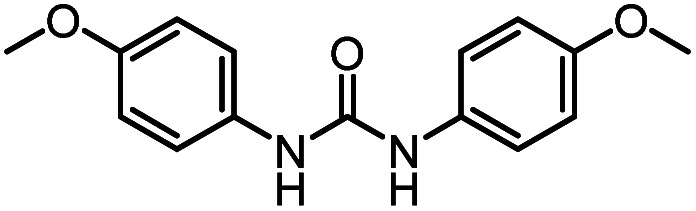	12	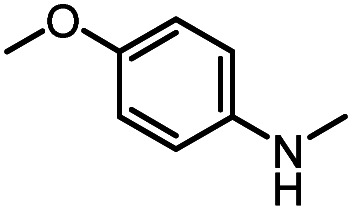	82	107
9	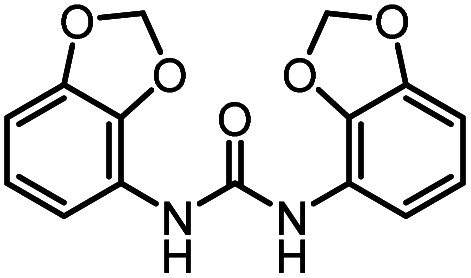	12^d^	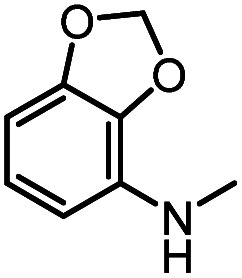	93	99
10	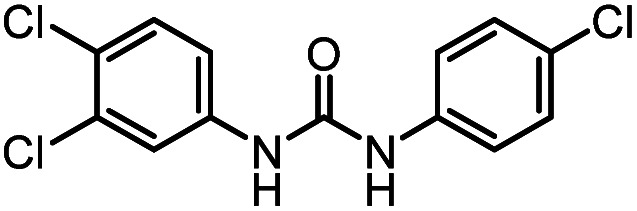	8	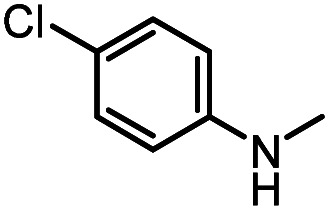	65	34
			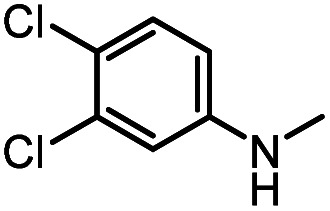	22	74

a Reaction conditions: substrate (2 mmol), (PPh_3_)_3_RuCl_2_ (1 mol%), triphos (1.5 mol%), KO^*t*^Bu (0.25 mol%), H_2_ (50 bar), THF (4 mL), 160 °C (bath temperature).

b Yield determined by GC using biphenyl as an internal standard. Identification of the products was also confirmed by GC-MS and ^1^H NMR; isolated yield in parentheses.

c NaHBEt_3_ was used instead of KO^*t*^Bu.

d 140 °C (bath temperature).

**Scheme 2:**
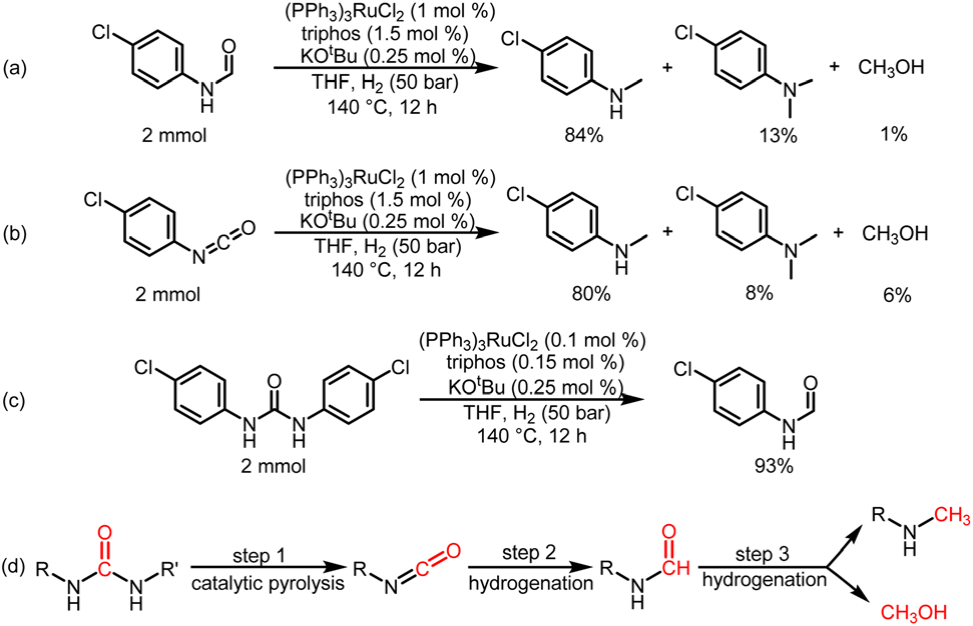
Control catalytic experiments. (a) Catalytic hydrogenation of *N*-(4-chlorophenyl)formamide in the presence of a Ru loading of 1% with 0.25 mol% KO^*t*^Bu. (b) Catalytic hydrogenation of 4-chlorphenylisocyanate in the presence of a Ru loading of 1% with 0.25 mol% KO^*t*^Bu. (c) Catalytic hydrogenation of 1,3-bis(4-chlorophenyl)urea in the presence of a low Ru loading of 0.1 mol% with 0.25 mol % KO^*t*^Bu. (d) Possible reaction pathway for hydrogenation of urea derivatives to six-electron reduction products.

To understand the reaction path for hydrogenation of urea derivatives to methylamines, some control experiments were carried out. Remarkably, performing the catalytic hydrogenation reaction using (PPh_3_)_3_RuCl_2_ (1 mol%) and triphos (1.5 mol%) in the absence of KO^*t*^Bu under H_2_ (50 bar) at 140 °C for 24 h in anhydrous tetrahydrofuran (THF), isocyanate and formamide were detected in addition to fully hydrogenated products (monomethylamine, dimethylamine and trace amounts of methanol) (Table 1, entry 8). In our previous studies,^33^ it was demonstrated that isocyanates were derived from the thermal decomposition of urea derivatives and were intermediates that form formamides. On this basis, 4-chlorophenyl isocyanate and *N*-(4-chlorophenyl)formamide were respectively used as substrates for hydrogenation experiments under identical reaction conditions, and these substrates were easily reduced to 4-chloro-*N*-methylaniline (Schemes 2a and b). Thus, according to preliminary experimental results, this reaction may be a sequential reaction of stepwise hydrogenation (Scheme 2d), in which urea derivatives underwent catalytic pyrolysis (step 1) and then the generated isocyanates were hydrogenated to formamides (step 2). They were eventually fully hydrogenated to form six-electron reduction products (step 3). In addition, the optimization experiment results showed that the conversion efficiency of the catalytic system with KO^*t*^Bu was significantly higher than that of the catalytic system without KO^*t*^Bu (Table 1). According to the results of control catalytic experiments in Schemes 2a (a Ru loading of 1% with 0.25 mol% KO^*t*^Bu) and 2c (a low Ru loading of 0.1 mol% with 0.25 mol% KO^*t*^Bu), it is speculated that the addition of KO^*t*^Bu will accelerate the reaction rate for hydrogenation of ureas to formamides, while the catalytic system in the absence of KO^*t*^Bu will promote the further hydrogenation of formamides to methylamine products.

**Fig. 1:**
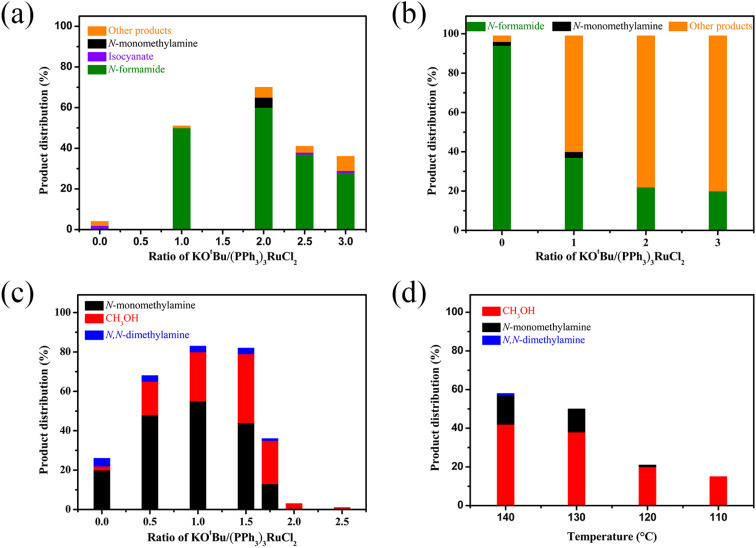
Reaction conditions: substrate (2 mmol), (PPh_3_)_3_RuCl_2_ (0.5 mol%), triphos (0.75 mol%), H_2_ (50 bar), THF (4 mL). (a) Substrate (1,3-bis(4-chlorophenyl)urea), 120 °C, 1 h; (b) substrate (4-chlorophenyl isocyanate), 120 °C, 1 h; (c) substrate (*N*-(4-chlorophenyl)formamide), 140 °C, 1 h; (d) substrate (*N*-(4-chlorophenyl)formamide), KO^*t*^Bu (1.75 equiv. relative to (PPh_3_)_3_RuCl_2_), 4 h.

We then attempted to gain insight into this interesting mechanism for switching the hydrogenation selectivity of urea derivatives by adjusting the amount of KO^*t*^Bu. Multiple control experiments were conducted *via* tuning the amount of KO^*t*^Bu to investigate its role. Increasing the KO^*t*^Bu loadings from 0 to 2 mol% while keeping the remaining conditions the same results in a gradual increase in the conversion of 1,3-bis(4-chlorophenyl)urea (Fig. 1a). In this reaction, when the KO^*t*^Bu loadings exceed 2.0 mol%, the hydrogenation efficiency of 1,3-bis(4-chlorophenyl)urea decreases slightly. Similarly, increasing the reaction temperature causes the conversion of 1,3-bis(4-chlorophenyl)urea to rise dramatically (Fig. S4†). It can be seen from Fig. 1b that the hydrogenation of isocyanate to formamide is a rapid hydrogenation process. With the increase of KO^*t*^Bu loadings, the selectivity of formamide decreases obviously and the formamidine byproduct was mainly formed (see the ESI† for details). Fig. 1a and b show that when the molar ratio of KO^*t*^Bu/(PPh_3_)_3_RuCl_2_ increases from 0 to 2.0, KO^*t*^Bu significantly improves the catalytic activity of the Ru catalyst in the thermal decomposition of urea into isocyanate, and the formation of isocyanate is rapidly converted to formamide. In the experiments for hydrogenation of formamide (Fig. 1c), the selectivity of methanol gradually increases as the molar ratio of KO^*t*^Bu/(PPh_3_)_3_RuCl_2_ increases from 0 to 2.0. When KO^*t*^Bu/(PPh_3_)_3_RuCl_2_ = 1.0, the catalytic activity for hydrogenation of formamide to methylamine and methanol is the best. However, when the molar ratio of KO^*t*^Bu/(PPh_3_)_3_RuCl_2_ is higher than 2.0, low hydrogenation activity of the catalyst system is observed, and only the strongest electrophilic reagent (isocyanate) can be reduced, while the weaker electrophilic reagent (formamide) will not be reduced. It is worth noting that isocyanate is difficult to convert in the absence of a Ru catalyst system (Fig. S1†). Thus, when the KO^*t*^Bu loadings are higher than 2 (2 equiv. relative to (PPh_3_)_3_RuCl_2_), it is conducive to the hydrogenation of the urea derivative to produce formamide and inhibits further hydrogenation of formamide. When the molar ratio of KO^*t*^Bu/(PPh_3_)_3_RuCl_2_ is lower than 2.0, high KO^*t*^Bu loadings are conducive to breaking the C–N bond to produce methanol, while low KO^*t*^Bu loadings tend to produce methylamine.

**Scheme 3:**
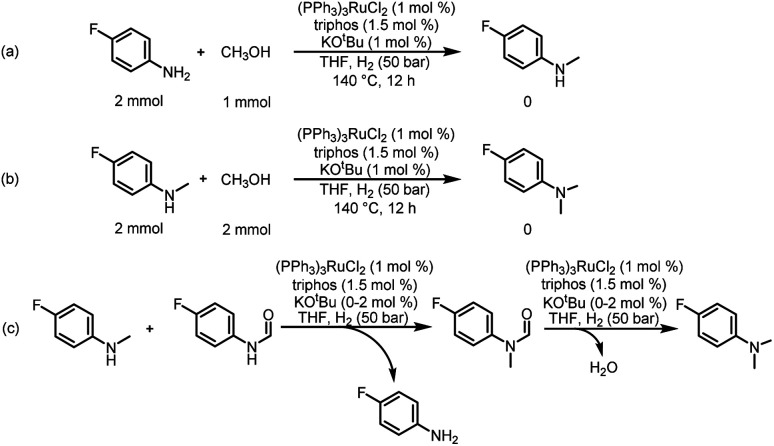
Possible pathway for the source of methyl groups. (a) Catalytic coupling of 4-fluoroaniline and methanol. (b) Catalytic coupling of 4-fluoro-*N*-methylaniline and methanol. (c) Possible reaction pathway for hydrogenation of urea derivatives to *N,N*-dimethylanilines.

**Scheme 4:**
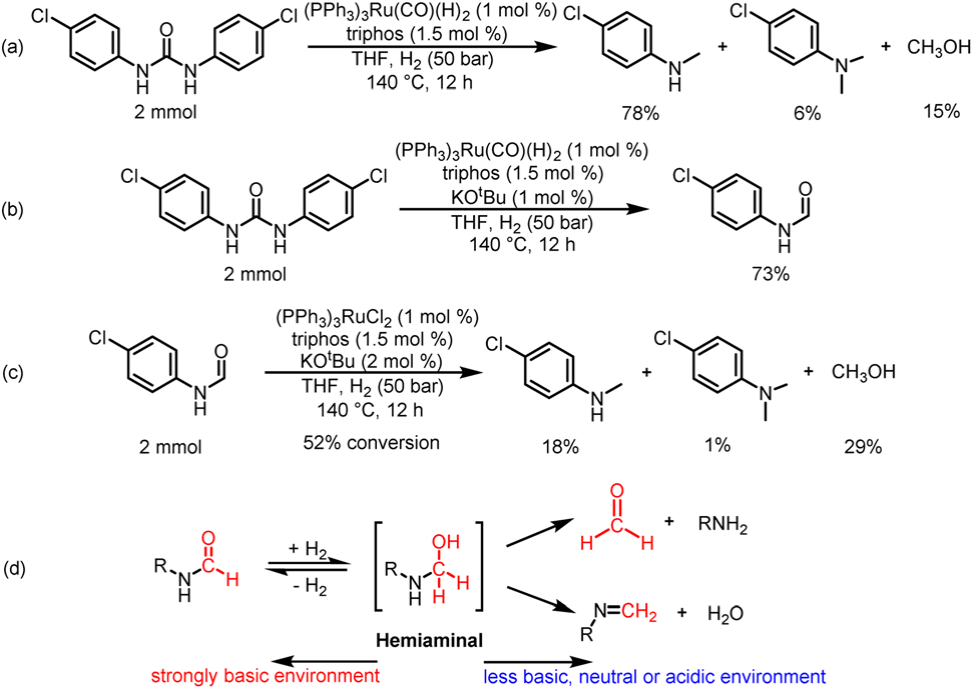
Mechanistic reactions. (a) Catalytic hydrogenation of 1,3-bis(4-chlorophenyl)urea with (PPh_3_)_3_Ru(CO)(H)_2_ as a metal precursor in the absence of KO^*t*^Bu. (b) Catalytic hydrogenation of 1,3-bis(4-chlorophenyl)urea with (PPh_3_)_3_Ru(CO)(H)_2_ as a metal precursor in the presence of 1 mol% KO^*t*^Bu. (c) Catalytic hydrogenation of *N*-(4-chlorophenyl)formamide in the presence of a Ru loading of 1 mol% with 2 mol% KO^*t*^Bu. (d) Transformation pathways of hemiaminal intermediate in acid–base environments.

In addition, from the perspective of kinetics, the proportion of products can be changed by adjusting the temperature. To further improve the selectivity for hydrogenation of formamide to methanol, we tried to reduce the reaction temperature to achieve this goal. Fig. 1d shows that with the decrease of temperature, the reaction rate slows down, while the selectivity of methanol gradually increases. This makes it possible to obtain highly selective methanol from urea derivatives by a two-step process (urea derivatives are first semi-hydrogenated to formamides, followed by hydrogenation of formamides to produce the highly selective methanol product). Moreover, it has been reported that methanol may be the source of methyl groups.^14,31,41^ Based on this, we conducted relevant experiments and found that the reaction occurred with difficulty when keeping the reaction conditions the same (Schemes 3a and b). Thus, the possibility of monomethylamine/dimethylamine formation by methanol and amine/methylamine can be ruled out. Furthermore, in combination with the hydrogenation experiments of formamide in Scheme 2a and Fig. 1, obvious dimethylamine byproducts can also be detected, thus dimethylamine may be caused by further reaction between monomethylamine and formamide (Scheme 4c).

In order to further understand the mechanism of this reaction, we first addressed the effect of base on the catalytic activity of the Ru-based catalyst system. According to the experimental results obtained, excess base may inhibit the catalytic activity of the catalyst system for the hydrogenation of formamide. This hypothesis is further supported by the results of an acid-base neutralization experiment: the excess HNTf_2_ neutralized the remaining base, leading to a significant change in the product from formamide to methylamine in the hydrogenation of 1,3-bis(4-chlorophenyl)urea (Fig. S5†). Unfortunately, HNTf_2_ may remain in the reaction solution. Lewis acids are known to activate amide bonds and promote nucleophilic addition to amides.^41–46^ Thus, it cannot be ruled out that residual HNTf_2_ promotes the hydrogenation of formamide.

Then, we discussed the identification and basic reactivity of Ru species for hydrogenation of urea derivatives. When a mixture of (PPh_3_)_3_RuCl_2_/triphos/2.5% KO^*t*^Bu/1,3-bis(4-chlorophenyl)urea was submitted to the catalytic conditions, the ESI-MS and NMR spectra of the reaction mixture indicated the formation of (triphos)Ru(CO)(H)_2_ complexes (see the ESI† for details). Importantly, the (triphos)Ru(CO)(H)_2_ species were also formed under a KO^*t*^Bu loading of 1%, which indicated that the catalytic species for catalytic hydrogenation of urea derivatives to two- and six-electron reduction products were associated with (triphos)Ru(CO)(H)_2_.^41,47^ When the (PPh_3_)_3_RuCl_2_/triphos/KO^*t*^Bu mixture was heated under catalytic conditions, the ESI-MS data of the obtained crude reaction solution indicated that (triphos)Ru(PPh_3_)(H)_2_ may be the precatalyst formed *in situ* (see the ESI† for details). After the addition of the substrate, the dissociative replacement of PPh_3_ occurred on the (triphos)Ru(PPh_3_)(H)_2_ complex by CO (it may be derived from decarbonylation of various compounds containing C–O bonds in the reaction solution) to form the (triphos)Ru(CO)(H)_2_ species *in situ*.^32,48,49^ Moreover, the formation of dimeric Ru-containing species [(triphos)Ru(μ-H)]_2_ was inhibited effectively. Based on these results, (PPh_3_)_3_Ru(CO)(H)_2_ was used to replace (PPh_3_)_3_RuCl_2_ as a metal precursor for the hydrogenation of 1,3-bis(4-chlorophenyl)urea in the absence of KO^*t*^Bu (Scheme 4a). Notably, the yield of the 4-chloro-*N*-methylaniline product was 78%, suggesting that (triphos)Ru(CO)(H)_2_ may be a catalytic intermediate for hydrogenation of urea derivatives to methylamines, and the coordination of the reaction substance is mainly through the transient loss of PPh_3_/CO. In addition, 1,3-bis(4-chlorophenyl)urea was successfully hydrogenated with (PPh_3_)_3_Ru(CO)(H)_2_ as a metal precursor in the presence of 1% KO^*t*^Bu. Significantly, the major product was *N*-(4-chlorophenyl)formamide with a 73% yield, and no significant six-electron reduction products were detected (Scheme 4b). Similarly, the addition of base inhibited the further transformation of formamide. This suggests that the base may have a dual role: (a) to abstract the chloride ligand from the metal precursor to produce a ruthenium complex of dihydride structure, and (b) to inhibit the hydrogenation of formamide to methanol and methylamine.

**Scheme 5:**
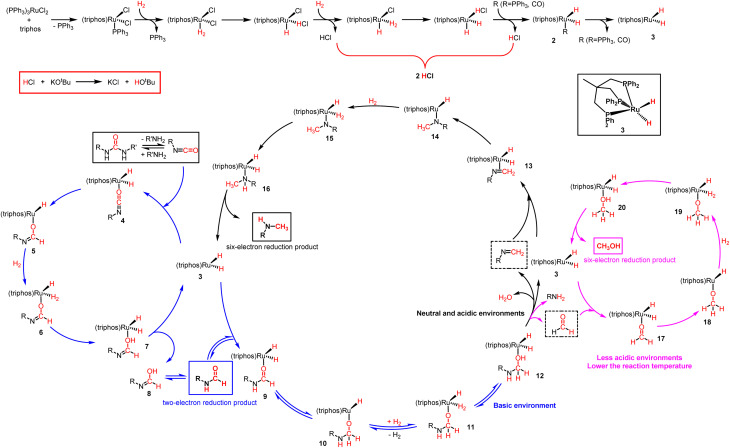
Proposed reaction mechanism for ruthenium-catalyzed hydrogenation of urea derivatives to formamides, methanol and monomethylamines.

Although we have some preliminary understanding of the dramatic switching selectivity from formamide to *N*-methylamine, it has not been rationalised appropriately. If the KO^*t*^Bu loading was less than 2 mol%, its main role was to abstract the Cl^−^ ligand on the metal precursor, which meant that the KO^*t*^Bu loading determines the content of catalytic active components. That is, the lower the KO^*t*^Bu loading, the poorer the catalytic efficiency. In fact, based on our experimental results, the outcome from the magic base loadings of 2 mol% and 1.5 mol% were very drastic (Table 1, entries 1 and 2). Further decrease in the base loading from 1.5 mol% to 0 mol% had no significant effect on the catalytic outcome (Table 1, entries 2–8). Unexpectedly, 1,3-bis(4-chlorophenyl)urea was able to convert well even in the absence of a base (Table 1, entry 8). By comparing the ESI-MS spectrum of the catalyst system lacking KO^*t*^Bu under a N_2_ atmosphere, it is obvious that additional catalytic intermediate (triphos)Ru(CO)(H)_2_ was detected in the reaction solution of the catalyst system in the absence of KO^*t*^Bu under a H_2_ atmosphere (Fig. S10 and S16†). Thus, the Cl^−^ ligand on the metal precursor may not be abstracted only through KO^*t*^Bu. H^−^ ligands on Ru metals can also be generated by H_2_ dissociation exchange (Fig. S17†). At a relatively high reaction temperature, the stable 6-coordinated 18-electron catalyst precursor opens the vacant site of the 5-coordinated 16-electron reactive intermediate, followed by H_2_ coordination. Subsequently, the hydrogen ligands undergo heterolytic dissociation, dissociating into H^+^ and H^−^, where H^−^ becomes a ligand, while H^+^ and Cl^−^ become HCl (Scheme 5),^50,51^ resulting in an acidic reaction solution. This conclusion is supported by the acid-base test results of the reaction solution (the reaction solution without KO^*t*^Bu is acidic, and the reaction solution in the presence of 2.5% KO^*t*^Bu is basic).

**Fig. 2:**
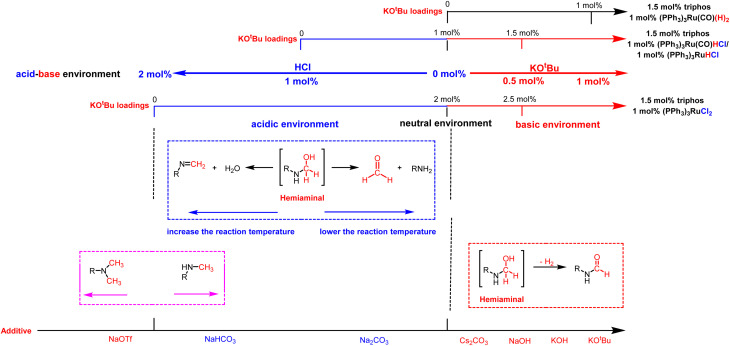
The effect of adding different amounts of bases and selecting different additives on the acidity–alkalinity of the reaction solution and selectivity of products.

In theory, the complete exchange of the Cl^−^ ligand on 1 mol% Ru metal by H^−^ produces 2 mol% HCl, which explains why the selectivity switches from formamide to *N*-methylated amine just by a change of 0.5 mol% KO^*t*^Bu (2 mol% to 1.5 mol%). The major role of base additive (KO^*t*^Bu) is to reduce the content of HCl in the reaction system and increase the pH value of the reaction solution (Scheme 5). When the KO^*t*^Bu loading is 2.5 mol%, the reaction solution is basic (in addition to neutralizing 2 mol% HCl, there is still a residual base), and the product is mainly formamide, a two-electron reduction product. When the KO^*t*^Bu loading is 2 mol%, the reaction solution approaches neutral and the product begins to be converted into six-electron reduction products (Scheme 4d), which is in line with the phenomenon reported by Verpoort and co-workers in the dehydrogenative amidation of alcohols and amines.^52^ In the absence of Cs_2_CO_3_, no amide formation was observed (C–O bond cleavage). Use of the base amount ranging from 1 to 3 mol% (a catalyst loading of 1 mol%) resulted in amide as a main product. When the KO^*t*^Bu loading is 1.5 mol%, the reaction solution becomes acidic and the products are dramatically and rapidly converted to six-electron reduction products. Previously, Cole–Hamilton and Leitner *et al.* also reported the dehydration of amides to form amines using the triphos/Ru catalyst system in the absence of additional additives and in the presence of an acid additive (MSA/HNTf_2_).^32,44,45^ Particularly, Leitner reported the formation of methyl aniline from diphenyl urea using Ru/triphos in the presence of an acid additive.^32^ In addition, we performed 1 mol% (PPh_3_)_3_RuHCl and (PPh_3_)_3_Ru(CO)HCl (metal Ru with one Cl^−^ ligand and one H^−^ ligand) substitution of (PPh_3_)_3_RuCl_2_ (metal Ru with two Cl^−^ ligands) as metal precursors for hydrogenation of urea derivatives, respectively. The experimental results were consistent with our expectations (Fig. S2†). When the KO^*t*^Bu loading is 1.5 mol% (higher than 1 mol%), the reaction product stays in the formamide stage. In the absence of KO^*t*^Bu, formamide continued to hydrogenate to yield six-electron reduction products (Fig. 2). Although there is some literature reporting that the formation of *N*-methylamine involves a dehydration step which is usually promoted by KO^*t*^Bu,^53,54^ this is a significant pathway in the hydrogen transfer reactions. In these catalytic reactions, the transfer of two hydrogen atoms may occur through the inner-sphere mechanism with deprotonation of the coordinated alcohol and β-H elimination of the resulting alkoxy group.^55^ Because the inner-sphere mechanism involves the coordination of the substrate to the metal fragment and then deprotonation with a Lewis base, additional bases are needed to facilitate the reaction to occur. The direct synthesis of imines from alcohols and amines reported by Milstein *et al.* does not require the addition of bases to achieve the dehydration step.^56^ Moreover, the formal deoxyhydrogenation of lactam reported by Kayaki can produce dehydrated products in the absence of bases.^57^

Based on these results, we examined the effect of base type in the presence of (PPh_3_)_3_RuCl_2_ (1 mol%), triphos (1.5 mol%), and base (2.5 mol%). 1,3-Bis(4-chlorophenyl)urea was hydrogenated under H_2_ (50 bar) at 140 °C for 12 h in THF as the benchmark reaction (Table 3). It was found that *N*-formamide was mainly formed with a strong base, and no significant six-electron reduction products were observed (Table 3, entries 1–4). Obviously, the yield of formamide with a Cs_2_CO_3_ loading of 2.5 mol% (Table 3, entry 3) was much lower than that of formamide with a low Cs_2_CO_3_ loading of 1.25 mol% (Table 3, entry 4). This may be caused by the excessive amount of base added, because neutralizing 2 mol% HCl requires about 1 mol% Cs_2_CO_3_ in theory, which is consistent with the result of relatively high KOH loadings (Table 3, entry 2). Importantly, the use of the weak bases Na_2_CO_3_ and NaHCO_3_ resulted in a substantial increase in the production of methylamine (Table 3, entries 5 and 7). When Na_2_CO_3_ and NaHCO_3_ loadings were increased, *N*-formamide yields were as high as 92% and 93%, and only a small amount of methylamine and methanol could be detected (Table 3, entries 6 and 8). It is worth noting that the formation of methylamine was also observed with sodium trifluoromethanesulfonate (NaOTf; an acid) as an additive, but the reaction rate was significantly slower (Table 3, entry 9). It is similar to the experimental results reported by Huynh *et al.*, which can selectively catalyze both dehydrogenative amidation and *N*-alkylation through coupling of alcohols with amines.^58^ Thus, the interesting selectivity for hydrogenation of urea derivatives to two- and six-electron reduction products may also be achieved by controlling the fate of a common hemiaminal intermediate (Scheme 4c).^55,59–64^ In a basic environment, the process of dehydrogenation of hemiaminal is promoted by the base, as this is a reversible process.^65^ The dehydrogenation rate (from hemiaminal to formamide) is greater than the hydrogenation rate (from formamide to hemiaminal), which is conducive to the formation of the formamide product.^52,58^ In contrast, in a neutral or acidic environment, hemiaminal further breaks the C–N bond to form formaldehyde or breaks the C–O bond to form an imine.^59^

**Table 3:** Hydrogenation of 1,3-bis(4-chlorophenyl)urea with different types of additive^a^

Entry	Additive	Yield (%) of 1a	Yield (%) of 1b	Yield (%) of 1c	Yield (%) of 1d
1	KOH	0	94	0	0
2^b^	KOH	0	42	0	0
3	Cs_2_CO_3_	0	33	<1	0
4^c^	Cs_2_CO_3_	0	87	<1	0
5^d^	Na_2_CO_3_	0	0	91	7
6	Na_2_CO_3_	2	92	3	0
7	NaHCO_3_	7	1	82	5
8^e^	NaHCO_3_	2	93	<1	0
9	NaOTf	0	22	4	46

a Reaction conditions: 1,3-bis(4-chlorophenyl)urea (2 mmol), (PPh_3_)_3_RuCl_2_ (1 mol%), triphos (1.5 mol%), additive (2.5 mol%), H_2_ (50 bar), THF (4 mL), 140 °C (bath temperature).

b KOH (4 mol%).

c Cs_2_CO_3_ (1.25 mol%).

d Na_2_CO_3_ (1.25 mol%).

e NaHCO_3_ (4 mol%).

Overall, we believe that selectivity in the formamide *versus* six-electron reduction products can be achieved by controlling the acidity and basicity of the reaction solution by adding different amounts of bases and choosing different additives (Fig. 2). In a basic environment (Scheme 4b), hemiaminal intermediates are more likely to release H_2_ to form formamide,^65,66^ while in a neutral environment (Scheme 4a) and acidic environment (Table 3, entry 9 and Fig. S5†), they eliminate H_2_O/amine to produce the corresponding imine/formaldehyde. When the acidity of the reaction solution was weakened or nearly neutral, the selectivity of formaldehyde was improved by eliminating amines with hemiaminal (Table 1 and Fig. 1c).^38,67^ Hemiaminal, on the other hand, helps eliminate the imine produced by H_2_O.^32,44,45^ It is worth noting that excessive acidity and alkalinity of the reaction environment can significantly reduce the efficiency of hydrogenation of urea derivatives (Fig. 1a and Table 3, entry 9). In addition, changing the reaction temperature can further improve the selectivity of the hemiaminal intermediates to eliminate H_2_O/amine to imine/formaldehyde (Fig. 1a). Within a certain reaction temperature range, increasing the reaction temperature is conducive to hemiaminal dehydration to methylamine, while lowering the reaction temperature can improve the selectivity of hemiaminal deamination to methanol.

Based on our experimental results and previous reports on the ruthenium-catalyzed hydrogenation of urea derivatives to formamides,^33^ we propose a plausible three competing catalytic cycles for hydrogenation of urea derivatives to *N*-formamides, *N*-monomethylamines, and methanol (Scheme 5). The precatalyst (triphos)Ru(PPh_3_)(H)_2_ (2) was prepared *in situ* by releasing HCl under H_2_ pressure using triphos and ruthenium precursor as raw materials. Then the PPh_3_ is dissociated to form the 16-electron dihydrogen complex 3, which is a reversible process. Catalytic thermal decomposition of the urea derivative occurs to eliminate R'NH_3_ to produce an isocyanate. Complex 3 is coordinated with the isocyanate to form substrate complex 4, which was then formed into hydride complex 5 by the classical migratory insertion step. The hydrogenolysis of the Ru–O bond is caused by the H_2_ molecular coordination to form complex 6, and the proton transfer to the oxygen through the adjacent H_2_ ligand leads to the formation of dihydride structure 7. Compound 8 is removed from 7 to regenerate active complex 3, and the released compound 8 undergoes tautomerism to form *N*-formamide as the product. *N*-formamide reacts with complex 3 to form complex 9, and neighboring H is transplanted and inserted to form 10. Complex 10 is coordinated with H_2_ to form 11, and protons are transferred to oxygen *via* the neighboring H_2_ ligand to form compound 12. In a basic environment, complex 12 terminates the subsequent reaction and dehydrogenates to formamide products *via* a reverse process.^65,66^ In contrast, the reaction continues, and the hemiaminal intermediate immediately loses water or the amine proceeds to hydrogenation. For formamide to methylamine conversion, complex 12 produces complex 13 by eliminating H_2_O.^56,68^ Then, complex 14 is formed by migration insertion, which is coordinated with hydrogen to form 15.^69^ The proton transfer to nitrogen *via* the adjacent H_2_ ligand (16) leads to the regeneration of the catalytically active species 3, releasing *N*-monomethylamine as a product. Similarly, for formamide to methanol conversion, complex 12 produces formaldehyde complex 17 at first by eliminating RNH_2_,^11,64,70^ then *via* migration insertion (18), coordination of an H_2_ molecule (19), and proton transfer (20), the catalytically active species 3 is finally regenerated and the methanol product is released.

## Conclusions

In summary, we developed an approach to control the product selectivity for hydrogenation of urea derivatives by changing the amount and type of additive in the *in situ* triphos-Ru catalyst system. The addition of different amounts and different types of additives is mainly used to change the acid–base environment of the reaction. In a basic environment, the urea derivatives undergo highly selective catalytic hydrogenation to formamides. However, if the added base is too high, it will seriously reduce the yield of formamides. In a neutral or an acidic environment, further hydrogenation of formamides was promoted to produce six-electron reduction products. Meantime, the selectivity of methanol in a less acidic or nearly neutral environment is higher than that in an acidic environment. Furthermore, lowering the reaction temperature can further improve the selectivity of methanol for hydrogenation of formamides, and increasing the temperature is conducive to the formation of methylamines. Our mechanism study shows that the active species for catalytic hydrogenation of urea derivatives to two- and six-electron reduction products are dihydrogen compound (triphos)Ru(H)_2_. It is due mainly to the addition of excess base that inhibited the further conversion of formamides. This study provides a green and mild synthetic route for the highly selective production of *N*-monomethylamine compounds, and develops an effective Ru-based catalyst system for the controlled synthesis of methylamines, formamides and methanol. Also, it may provide new insights into the formylation and alkylation of amines using CO_2_ and H_2_.

## Data availability

All data needed to evaluate the conclusions in the paper are present in the paper and/or the ESI.† And all data can be obtained from the authors.

## Author contributions

H. L. conceived the project and supervised the work with Y. W. and J. Y. J. Z. performed the catalysis experiments and wrote the manuscript. All authors discussed the results and contributed to the final manuscript.

## Conflicts of interest

There are no conflicts to declare.

## Supplementary Material

SC-015-D3SC05674K-s001
