# Supplementary material for: Switching the hydrogenation selectivity of urea derivatives via subtly tuning the amount and type of additive in the catalyst system
Source: Chem Sci. 2023 Dec 23;15(6):2089–99. doi: 10.1039/d3sc05674k (PMC10848806; doi:10.1039/d3sc05674k)
Supplement: SC-015-D3SC05674K-s001 [file SC-015-D3SC05674K-s001.pdf]

Electronic Supplementary Material (ESI) for Chemical Science.
This journal is © The Royal Society of Chemistry 2023

# Supporting Information

# Switching Hydrogenation Selectivity of Urea Derivatives via Tuning the Amount and Type of Additive in the Catalyst System

Jun Zhu, Yongtao Wang, Jia Yao and Haoran Li

Department of Chemistry, ZJU-NHU United R&D Center, Zhejiang University, Hangzhou 310027, China.

# Table of Contents

1. General Experimental ........ 3

2. Procedure for the catalytic hydrogenation of urea derivatives ........ 3

3. Further experimental data ........ 4

(a) Hydrogenation experiment ........ 4

(b) Acid-base neutralization experiment ........ 7

(c) Hydrogenation experiment of *N*-(4-chlorophenyl)formamide ........ 8

4. Identification of precatalyst before the catalytic hydrogenation of 1,3-bis(4-chlorophenyl)urea ........ 9

(a) $(PPh_3)_3RuCl_2$/triphos/$KO^tBu$ in a 1:1.5:2.5 molar ratio in THF ........ 9

(b) $(PPh_3)_3RuCl_2$/triphos/$KO^tBu$ in a 1:1.5:1 molar ratio in THF ........ 10

(c) $(PPh_3)_3RuCl_2$/triphos/$KO^tBu$/$HNTf_2$ in a 1:1.5:2.5:1 molar ratio in THF ........ 11

(d) $(PPh_3)_3RuCl_2$/triphos in a 1:1.5 molar ratio under $N_2$ atmosphere in THF ........ 12

5. Identification of catalytic intermediate after the catalytic hydrogenation of 1,3-bis(4-chlorophenyl)urea ........ 13

(a) $(PPh_3)_3RuCl_2$/triphos/$KO^tBu$/1,3-bis(4-chlorophenyl)urea in a 1:1.5:2.5:100 molar ratio in THF ........ 13

(b) $(PPh_3)_3RuCl_2$/triphos/$KO^tBu$/1,3-bis(4-chlorophenyl)urea in a 1:1.5:1:100 molar ratio in THF ........ 15

(c) $(PPh_3)_3Ru(CO)(H)_2$/triphos/$KO^tBu$/1,3-bis(4-chlorophenyl)urea in a 1:1.5:1:100 molar ratio in THF ........ 16

(d) $(PPh_3)_3Ru(CO)(H)_2$/triphos /1,3-bis(4-chlorophenyl)urea in a 1:1.5:100 molar ratio in THF ........ 17

(e) $(PPh_3)_3RuCl_2$/triphos/1,3-bis(4-chlorophenyl)urea in a 1:1.5:100 molar ratio in THF ........ 18

6. Analysis of organic compounds ........ 20

(a) GC-MS Data of Products ........ 20

(b) $^1H$ NMR Data of Products ........ 25

(c) NMR Data of *N*-methylaniline Products ........ 31

References ........ 35

## 1. General Experimental

All experiments were carried out under an atmosphere of purified nitrogen in a Vacuum Atmospheres glove box. All solvents were reagent grade or better. Deuterated solvents were used as received. All solvents were degassed with argon and kept in the glove box over 4Å molecular sieves. All the chemicals used in the catalytic reactions and authentic samples of the product (*N*-methylamines and amines) are commercially available.

NMR spectra were recorded using Bruker-500 instrument. $^1H$ NMR chemical shifts are reported in ppm downfield from tetramethylsilane and referenced to the residual signals of an appropriate deuterated solvent. Mass spectra were recorded on Agilent 6545 Q-TOF, using Electro Spray Ionization (ESI) mode. GC-MS was carried out on a Shimadzu GC/MS-QP2010 system (Shimadzu, Germany), carried out using a DB-5 column (30 m × 0.25 mm × 0.25 μm film thickness), and helium as carrier gas. GC analysis were obtained on GC-2010 (Shimadzu, Japan) and carried out using a HP-1 column (30 m × 0.25 mm × 0.25 μm film thickness) using biphenyl as an internal standard. The quantitative analysis of the products (formamides and amines) was performed by comparison with the corresponding authentic samples (commercially purchased). Inlets: 280 $^oC$; Detector: FID 300 $^oC$; Carrier Gas: $N_2$; Flow: 1 mL/min; Oven: 40 $^oC$, hold 7 min; 5 $^oC$/min to 80 $^oC$, hold 3 min; 2 $^oC$/min to 100 $^oC$, hold 3 min; 20 $^oC$/min to 300 $^oC$, hold 3 min.

## 2. Procedure for the catalytic hydrogenation of urea derivatives

In a $N_2$ glove box, 0.01 mmol of the ruthenium precursor, 0.015 mmol of 1,1,1-tris(diphenylphosphinomethyl)ethane and 0.0025 mmol of $KO^tBu$ were added in 4 mL of THF to a 50 mL autoclave (Anhui CHEM$^N$ Instrument Co., Ltd). This mixture was stirred for 5 min, then 2 mmol urea derivative was added to it. Remove the sealed autoclave from the glove box, rinse it four times with hydrogen gas (Pressure to 50 bar, release to 2 bar, cycle four times) and pressurize it to the specified pressure, and heat it with stirring at the specified temperature. After the reaction, the steel autoclave was cooled in an ice-bath for 30 min and slowly depressurized. The biphenyl (internal standard) was added to the cold solution and then filtered through Celite, and the solution was analyzed by GC/GC-MS and $^1H$ NMR spectroscopy.

## 3. Further experimental data

### (a) Hydrogenation experiment

In a $N_2$ glove box, 2 mmol chlorophenylisocyanate was added in 4 mL of THF to a 50 mL autoclave. Remove the sealed autoclave from the glove box, rinse it four times with $H_2$ (Pressure to 50 bar, release to 2 bar, cycle four times) and pressurize it to the specified pressure, and heat it with stirring at the specified temperature. After the reaction, the steel autoclave was cooled in an ice-bath for 30 min and slowly depressurized. The biphenyl (internal standard) was added to the cold solution and then filtered through Celite, and the solution was analyzed by GC.

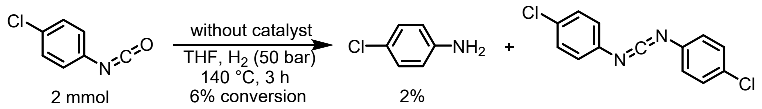


**Figure S1.** Hydrogenation experiment of isocyanate.

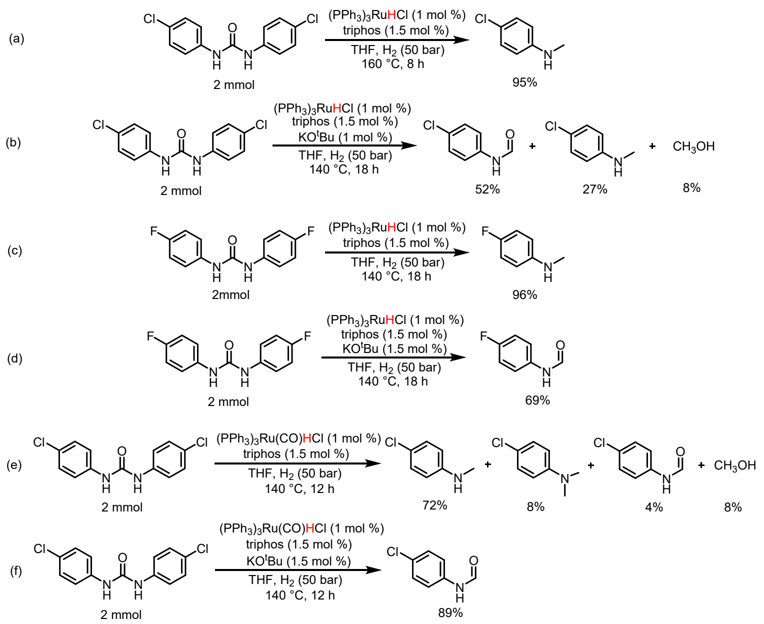


**Figure S2.** $(PPh_3)_3RuHCl$ and $(PPh_3)_3Ru(CO)HCl$ replace $(PPh_3)_3RuCl_2$ as metal precursors for hydrogenation of urea derivatives.

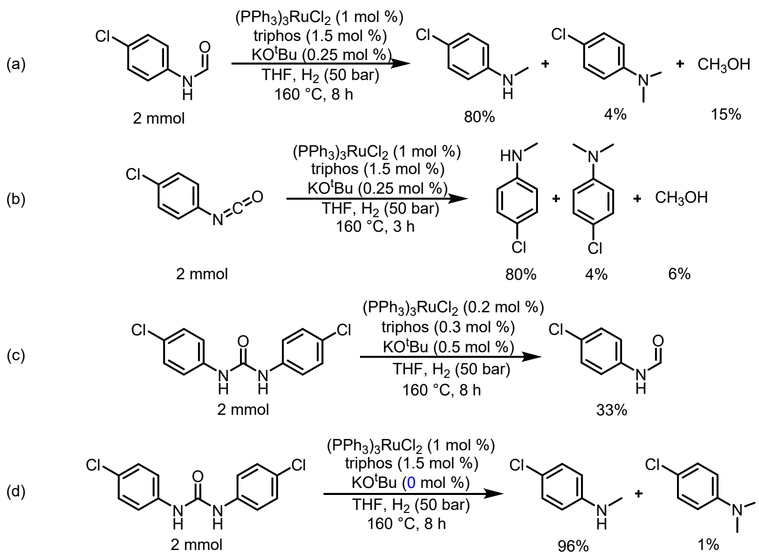


**Figure S3.** The control experiment was carried out at 160 °C.

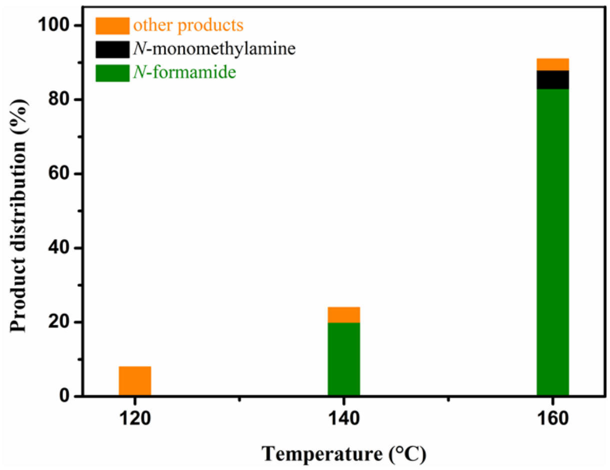


**Figure S4.** Reaction conditions: substrate (1,3-bis(4-chlorophenyl)urea; 2 mmol), $(PPh_3)_3RuCl_2$ (0.5 mol %), triphos (0.75 mol %), $H_2$ (50 bar), THF (4 mL), 1 h.

**(b) Acid-base neutralization experiment**

In a $N_2$ glove box, 0.01 mmol of the ruthenium precursor, 0.015 mmol of 1,1,1-tris(diphenylphosphinomethyl)ethane and 0.025 mmol of $KO^tBu$ were added in 4 mL of THF to a 50 mL autoclave. Remove the sealed autoclave from the glove box, rinse it four times with hydrogen gas (Pressure to 50 bar, release to 2 bar, cycle four times) and pressurize it to the specified pressure, and heat it with stirring at the specified temperature. After reaction 2h, the steel autoclave was cooled in an ice-bath for 30 min. Then, 0.01 mmol of $HNTf_2$ was added to it were added in catalyst solution in a $N_2$ glove box. This mixture was stirred for 5 min, then 2 mmol of 1,3-bis(4-chlorophenyl)urea was added to it. Remove the sealed autoclave from the glove box, rinse it four times with hydrogen gas (Pressure to 50 bar, release to 2 bar, cycle four times) and pressurize it to the specified pressure, and heat it with stirring at the specified temperature. After reaction 12h, the steel autoclave was cooled in an ice-bath for 30 min and slowly depressurized. The biphenyl (internal standard) was added to the cold solution and then filtered through Celite, and the solution was analyzed by GC.

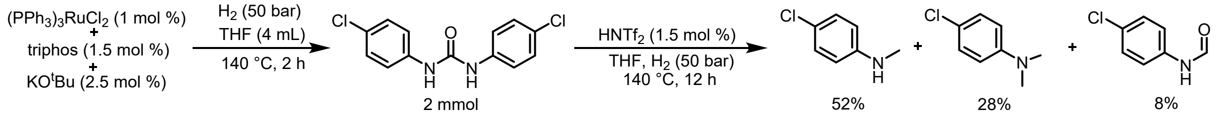


**Figure S5.** $HNTf_2$ was used to neutralize excess $KO^tBu$ in the reaction solution.

**(c) Hydrogenation experiment of *N*-(4-chlorophenyl)formamide**

In a $N_2$ glove box, 0.01 mmol of the ruthenium precursor and 0.01 mmol of $KO^tBu$ were added in 4 mL of THF to a 50 mL autoclave. This mixture was stirred for 5 min, then 2 mmol *N*-(4-chlorophenyl)formamide was added to it. Remove the sealed autoclave from the glove box, rinse it four times with hydrogen gas (Pressure to 50 bar, release to 2 bar, cycle four times) and pressurize it to the specified pressure, and heat it with stirring at the specified temperature. After reaction 8h, the steel autoclave was cooled in an ice-bath for 30 min and slowly depressurized. The biphenyl (internal standard) was added to the cold solution and then filtered through Celite, and the solution was analyzed by GC.

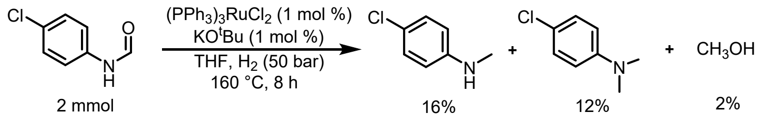


**Figure S6.** Hydrogenation experiment of *N*-(4-chlorophenyl)formamide in the absence triphos.

## 4. Identification of precatalyst before the catalytic hydrogenation of 1,3-bis(4-chlorophenyl)urea

### (a) $(PPh_3)_3RuCl_2$/triphos/KO$^t$Bu in a 1:1.5:2.5 molar ratio in THF

A 50 mL autoclave was charged with a solution of 1,1,1-tris(diphenylphosphinomethyl)ethane (0.03 mmol), $(PPh_3)_3RuCl_2$ (0.02 mmol) and $^t$BuOK (0.05 mmol) in 4.0 mL THF. The autoclave was pressurized with 50 bar $H_2$ and heated for 4 h at 140 °C. After the reaction, the autoclave was cooled in an ice-bath for 30 min and vented in a glove box. 0.1 mL of the solution filtered with Celite was sealed in a vial for ESI-MS test.

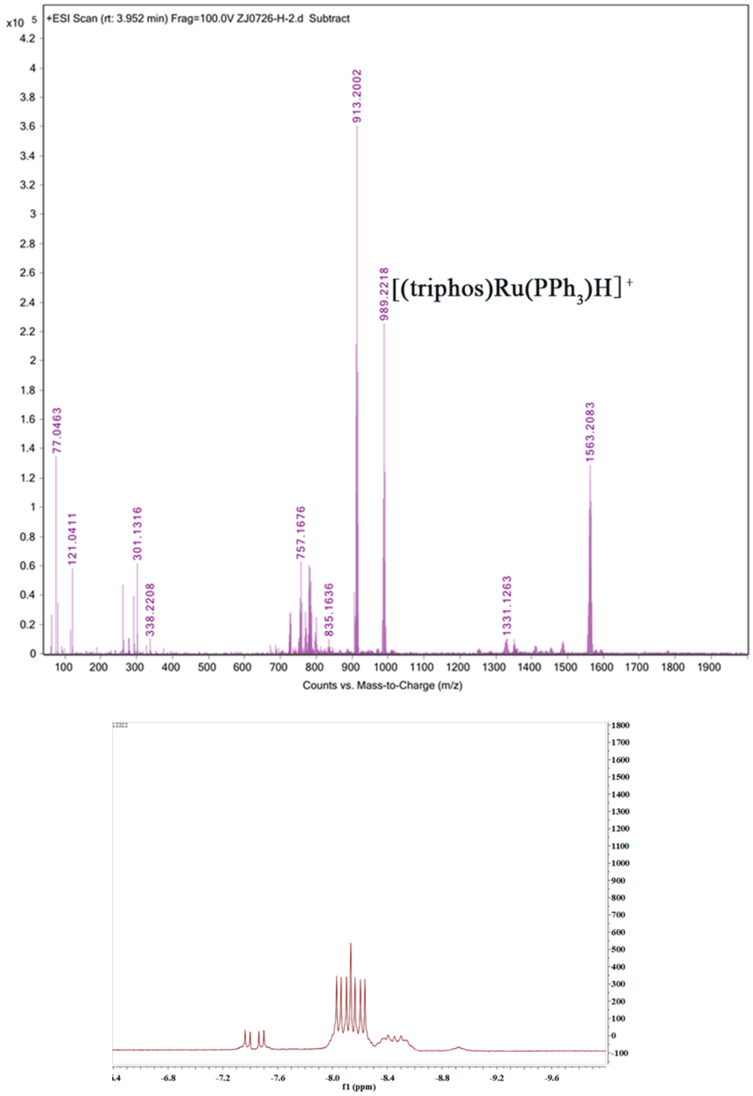


**Figure S7.** ESI(+) mass spectrum and $^1$H NMR (500 MHz, d$^2$-dichlormethane) spectrum.

**(b) $(PPh_3)_3RuCl_2$/triphos/KO$^t$Bu in a 1:1.5:1 molar ratio in THF**

A 50 mL autoclave was charged with a solution of 1,1,1-tris(diphenylphosphinomethyl)ethane (0.03 mmol), $(PPh_3)_3RuCl_2$ (0.02 mmol) and $^t$BuOK (0.02 mmol) in 4.0 mL THF. The autoclave was pressurized with 50 bar $H_2$ and heated for 4 h at 140 °C. After the reaction, the autoclave was cooled in an ice-bath for 30 min and vented in a glove box. 0.1 mL of the solution filtered with Celite was sealed in a vial for ESI-MS test.

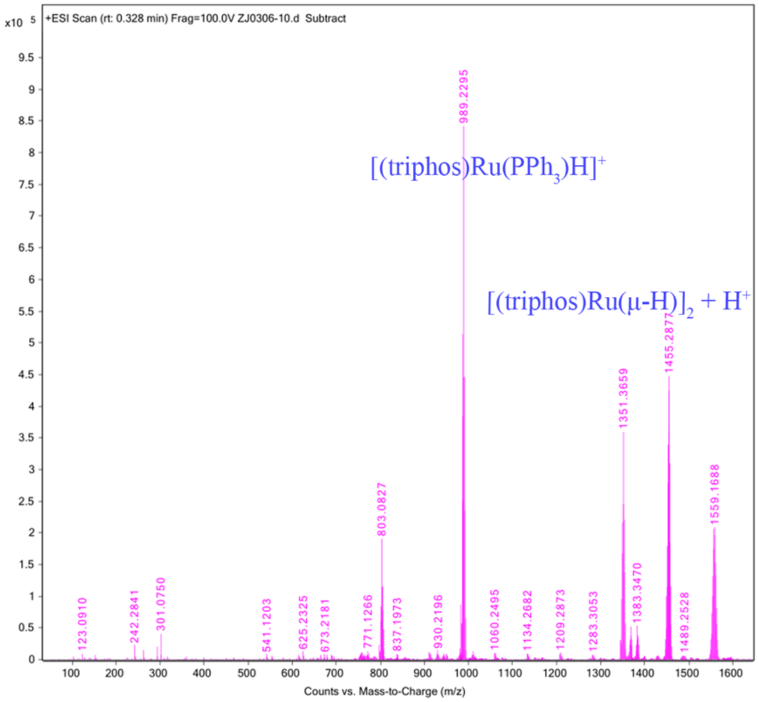


**Figure S8.** ESI(+) mass spectrum.

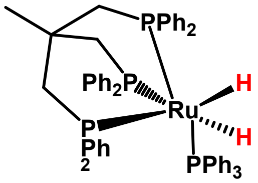


**ESI-MS (MeOH)** $C_{59}H_{55}P_4Ru^+$ (M-H) Calc.: 989.2 Found: 989.2 m/z.

**(c) $(PPh_3)_3RuCl_2$/triphos/$KO^tBu$/$HNTf_2$ in a 1:1.5:2.5:1 molar ratio in THF**

In a $N_2$ glove box, 0.01 mmol of the ruthenium precursor, 0.015 mmol of 1,1,1-tris(diphenylphosphinomethyl)ethane and 0.025 mmol of $KO^tBu$ were added in 4 mL of THF to a 50 mL autoclave. Remove the sealed autoclave from the glove box, rinse it four times with hydrogen gas (Pressure to 50 bar, release to 2 bar, cycle four times) and pressurize it to the specified pressure, and heat it with stirring at the specified temperature. After reaction 2h, the steel autoclave was cooled in an ice-bath for 30 min. Then, 0.01 mmol of $HNTf_2$ was added to it were added in catalyst solution in a $N_2$ glove box. Remove the sealed autoclave from the glove box, rinse it four times with hydrogen gas (Pressure to 50 bar, release to 2 bar, cycle four times) and pressurize it to the specified pressure, and heat it with stirring at the specified temperature. After reaction 12h, the autoclave was cooled in an ice-bath for 30 min and vented in a glove box. 0.1 mL of the solution filtered with Celite was sealed in a vial for ESI-MS test.

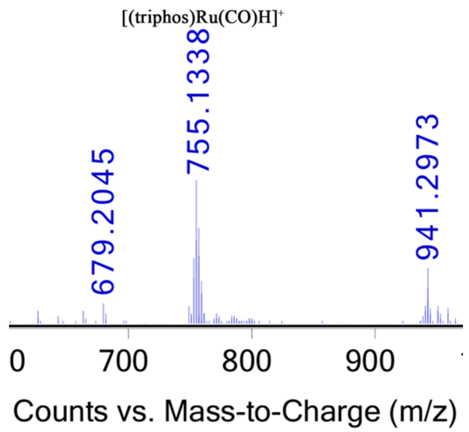


**ESI-MS (MeOH)** $C_{42}H_{40}OP_3Ru^+$ (M-H) Calc.: 755.1 Found: 755.1 m/z.

**Figure S9.** ESI(+) mass spectrum.

**(d) $(PPh_3)_3RuCl_2$/triphos in a 1:1.5 molar ratio under $N_2$ atmosphere in THF**

In a $N_2$ glove box, 0.01 mmol of the ruthenium precursor and 0.015 mmol of 1,1,1-tris(diphenylphosphinomethyl)ethane were added in 4 mL of THF to a 50 mL autoclave. This mixture was stirred for 5 min, then 2 mmol 1,3-bis(4-chlorophenyl)urea was added to it. Remove the sealed autoclave from the glove box, and heated for 12 h at 140 °C. After the reaction, the autoclave was cooled in an ice-bath for 30 min and vented in a glove box. 0.1 mL of the solution filtered with Celite was sealed in a vial for ESI-MS test.

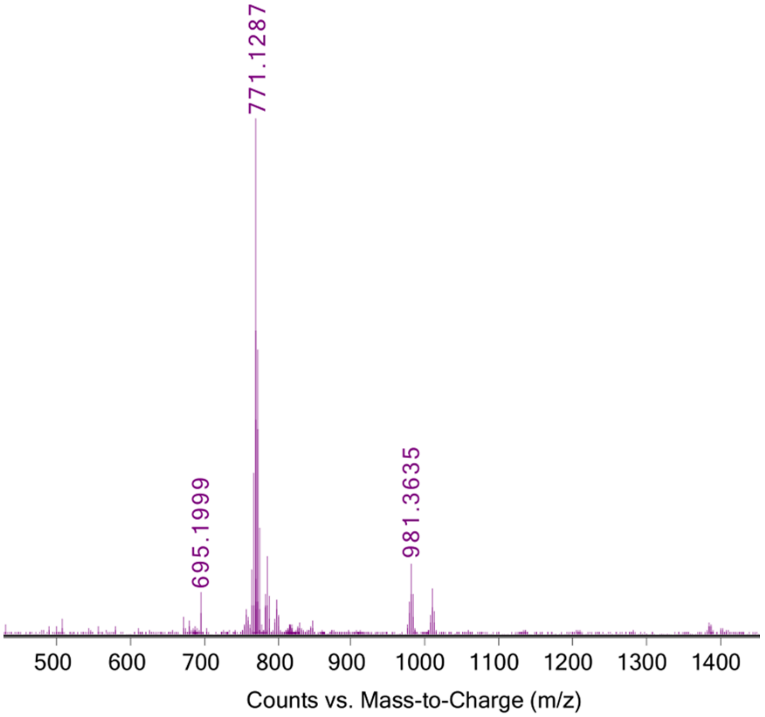


**Figure S10.** ESI(+) mass spectrum.

## 5. Identification of catalytic intermediate after the catalytic hydrogenation of 1,3-bis(4-chlorophenyl)urea

#### (a) $(PPh_3)_3RuCl_2$/triphos/KO$^t$Bu/1,3-bis(4-chlorophenyl)urea in a 1:1.5:2.5:100 molar ratio in THF

In a $N_2$ glove box, 0.01 mmol of the ruthenium precursor, 0.015 mmol of 1,1,1-tris(diphenylphosphinomethyl)ethane and 0.025 mmol of $^t$BuOK were added in 4 mL of THF to a 50 mL autoclave. This mixture was stirred for 5 min, then 2 mmol 1,3-bis(4-chlorophenyl)urea was added to it. Remove the sealed autoclave from the glove box, rinse it four times with hydrogen gas (Pressure to 50 bar, release to 2 bar, cycle four times) and pressurize it to the specified pressure, and heat it with stirring at the specified temperature. After the reaction, the steel autoclave was cooled in an ice-bath for 30 min and slowly depressurized. Afterwards, THF was slowly evaporated and the product precipitated form the concentrated reaction mixture which was analysed by NMR. Data is in accordance with literature.[1-3]

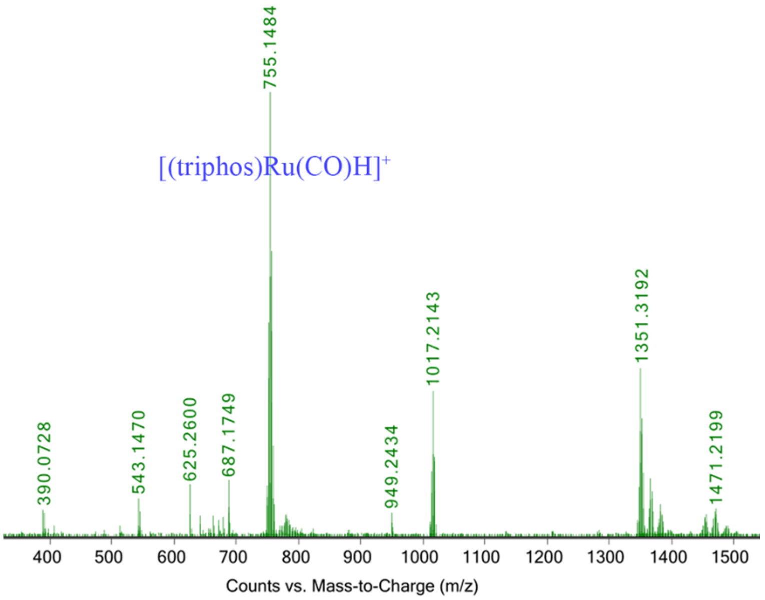


**Figure S11.** ESI(+) mass spectrum of the reaction crude.

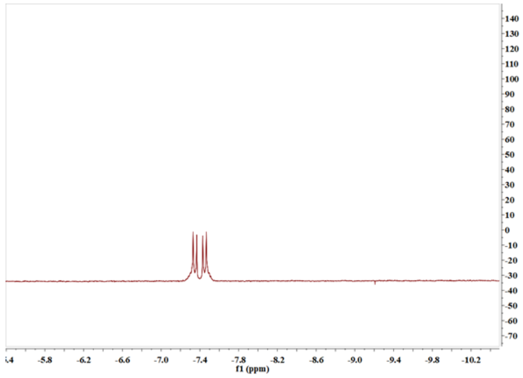


**Figure S12.** $^1$H NMR (500 MHz) spectrum in d$^2$-dichlormethane.

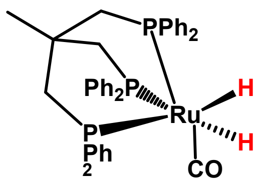


**ESI-MS (MeOH)** $C_{42}H_{40}OP_3Ru^+$ (M-H) Calc.: 755.1 Found: 755.1 m/z.

**(b) $(PPh_3)_3RuCl_2$/triphos/$KO^tBu$/1,3-bis(4-chlorophenyl)urea in a 1:1.5:1:100 molar ratio in THF**

In a $N_2$ glove box, 0.01 mmol of the ruthenium precursor, 0.015 mmol of 1,1,1-tris(diphenylphosphinomethyl)ethane and 0.01 mmol of $^tBuOK$ were added in 4 mL of THF to a 50 mL autoclave. This mixture was stirred for 5 min, then 2 mmol 1,3-bis(4-chlorophenyl)urea was added to it. Remove the sealed autoclave from the glove box, rinse it four times with hydrogen gas (Pressure to 50 bar, release to 2 bar, cycle four times) and pressurize it to the specified pressure, and heat it with stirring at the specified temperature. After the reaction, the autoclave was cooled in an ice-bath for 30 min and vented in a glove box. 1 mL of the solution filtered with Celite was sealed in a vial for ESI-MS test.

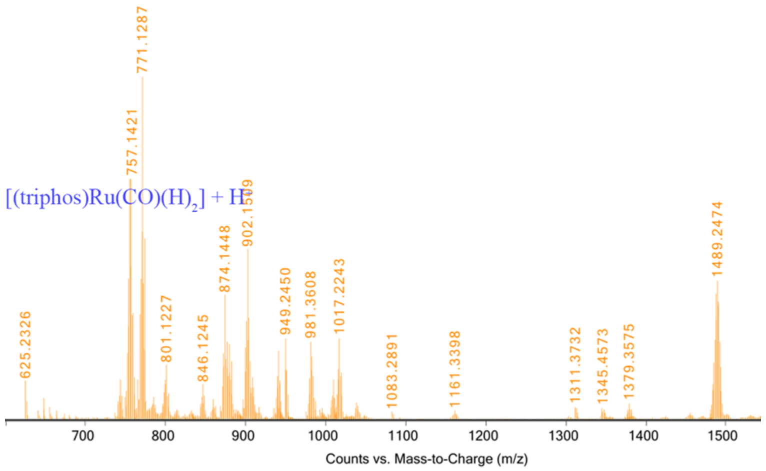


**Figure S13.** ESI(+) mass spectrum of the reaction crude.

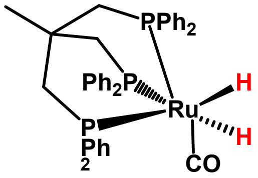


**ESI-MS (MeOH)** $C_{42}H_{42}OP_3Ru$ ($M + H^+$) Calc.: 757.1 Found: 757.1 m/z.

**(c) $(PPh_3)_3Ru(CO)(H)_2$/triphos/KO$^t$Bu/1,3-bis(4-chlorophenyl)urea in a 1:1.5:1:100 molar ratio in THF**

In a $N_2$ glove box, 0.01 mmol of $(PPh_3)_3Ru(CO)(H)_2$, 0.015 mmol of 1,1,1-tris(diphenylphosphinomethyl)ethane and 0.01 mmol of $^t$BuOK were added in 4 mL of THF to a 50 mL autoclave. This mixture was stirred for 5 min, then 2 mmol 1,3-bis(4-chlorophenyl)urea was added to it. Remove the sealed autoclave from the glove box, rinse it four times with hydrogen gas (Pressure to 50 bar, release to 2 bar, cycle four times) and pressurize it to the specified pressure, and heat it with stirring at the specified temperature. After reaction 12h, the autoclave was cooled in an ice-bath for 30 min and vented in a glove box. 1 mL of the solution filtered with Celite was sealed in a vial for ESI-MS test.

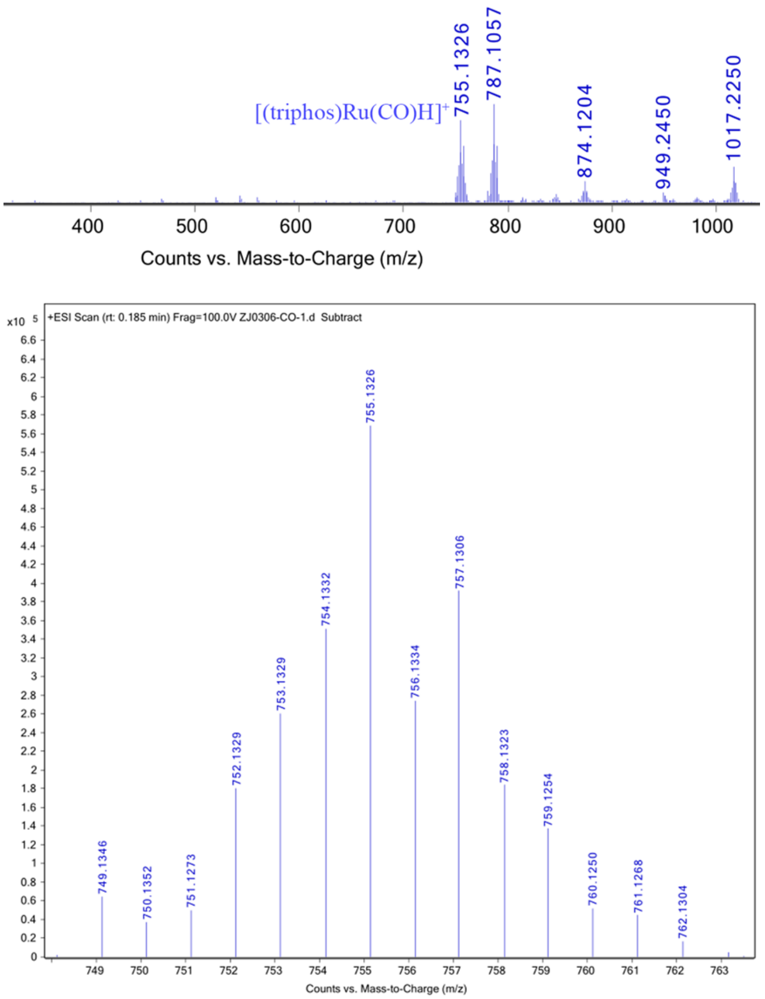


**Figure S14.** ESI(+) mass spectrum of the reaction crude.

**(d) $(PPh_3)_3Ru(CO)(H)_2$/triphos /1,3-bis(4-chlorophenyl)urea in a 1:1.5:100 molar ratio in THF**

In a $N_2$ glove box, 0.01 mmol of $(PPh_3)_3Ru(CO)(H)_2$ and 0.015 mmol of 1,1,1-tris(diphenylphosphinomethyl)ethane were added in 4 mL of THF to a 50 mL autoclave. This mixture was stirred for 5 min, then 2 mmol 1,3-bis(4-chlorophenyl)urea was added to it. Remove the sealed autoclave from the glove box, rinse it four times with hydrogen gas (Pressure to 50 bar, release to 2 bar, cycle four times) and pressurize it to the specified pressure, and heat it with stirring at the specified temperature. After reaction 12h, the autoclave was cooled in an ice-bath for 30 min and vented in a glove box. 1 mL of the solution filtered with Celite was sealed in a vial for ESI-MS test.

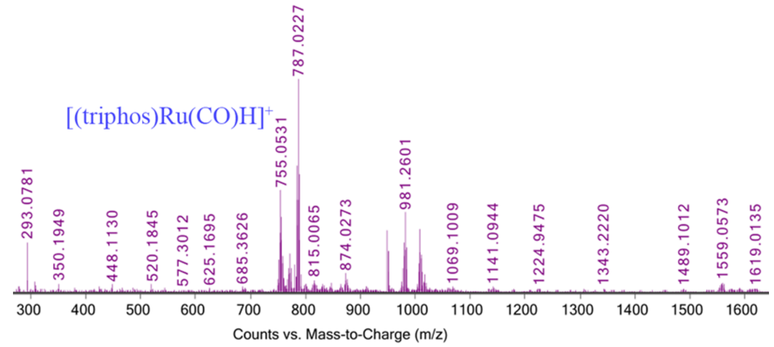


**Figure S15.** ESI(+) mass spectrum of the reaction crude.

**(e) $(PPh_3)_3RuCl_2$/triphos/1,3-bis(4-chlorophenyl)urea in a 1:1.5:100 molar ratio in THF**

In a $N_2$ glove box, 0.01 mmol of $(PPh_3)_3RuCl_2$ and 0.015 mmol of 1,1,1-tris(diphenylphosphinomethyl)ethane were added in 4 mL of THF to a 50 mL autoclave. This mixture was stirred for 5 min, then 2 mmol 1,3-bis(4-chlorophenyl)urea was added to it. Remove the sealed autoclave from the glove box, rinse it four times with hydrogen gas (Pressure to 50 bar, release to 2 bar, cycle four times) and pressurize it to the specified pressure, and heat it with stirring at the specified temperature. After reaction 12h, the autoclave was cooled in an ice-bath for 30 min and vented in a glove box. 1 mL of the solution filtered with Celite was sealed in a vial for ESI-MS test.

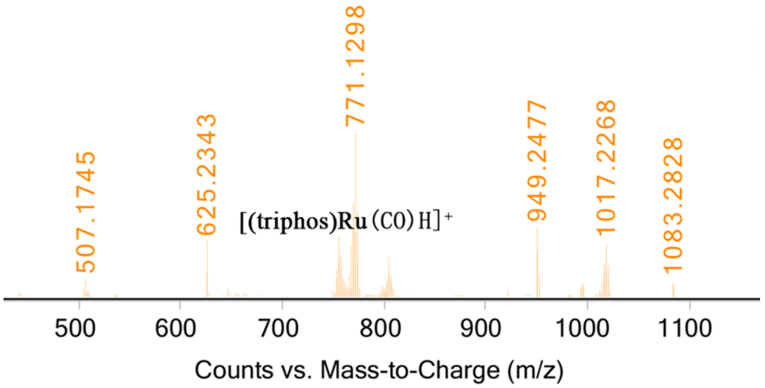


**ESI-MS (MeOH)** $C_{42}H_{40}OP_3Ru^+$ (M-H) Calc.: 755.1 Found: 755.1 m/z.

**Figure S16.** ESI(+) mass spectrum of the reaction crude.

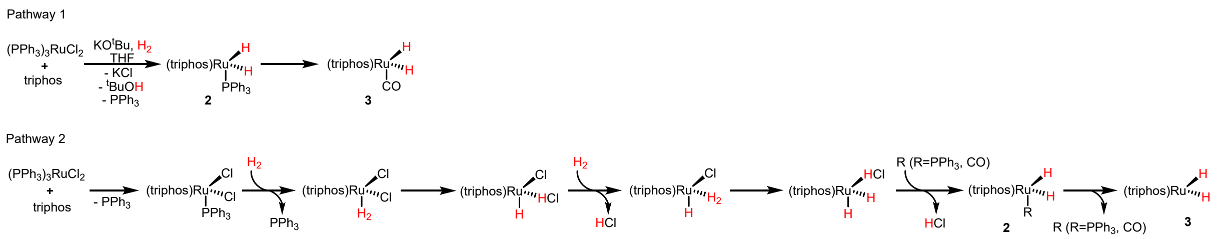


**Figure S17.** Possible formation paths of $(PPh_3)_3Ru(CO)(H)_2$ catalytic intermediate.

## 6. Analysis of organic compounds

### (a) GC-MS Data of Products

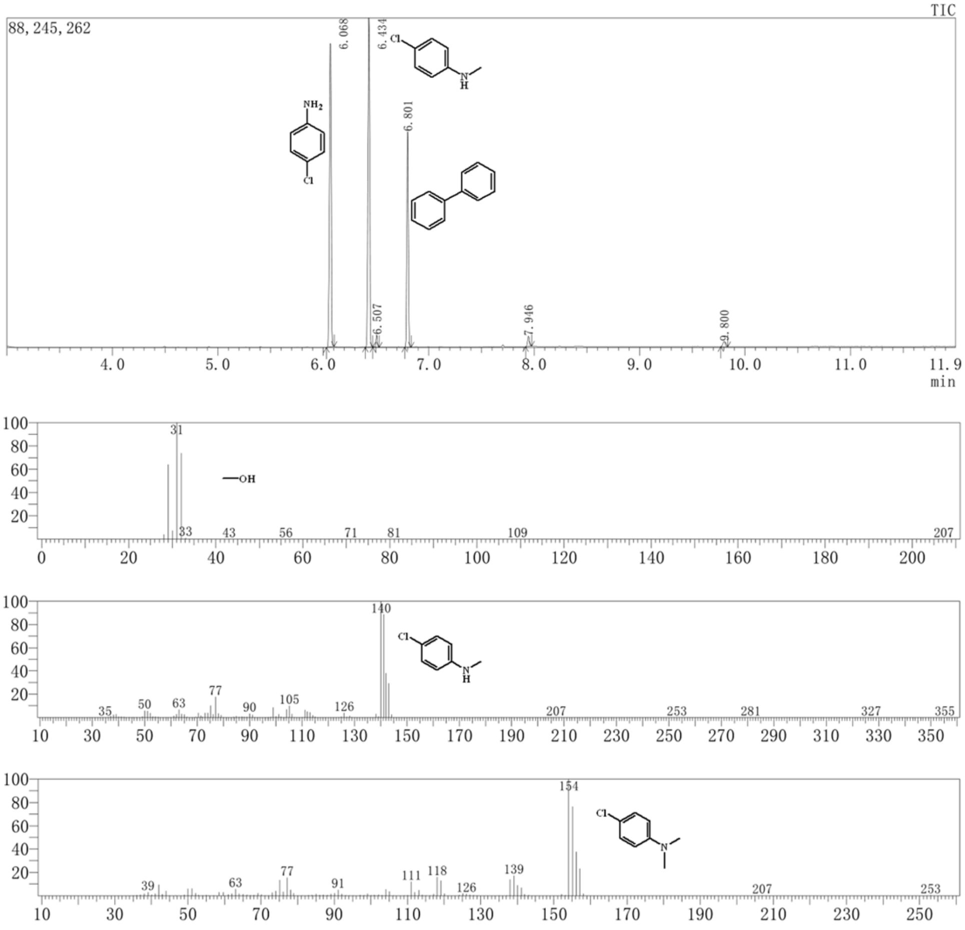

**Figure S18.** GC-MS spectrum of the crude reaction mixture of hydrogenation of 1,3-bis(4-chlorophenyl)urea (Table 1, entry 11) with biphenyl as internal standard.

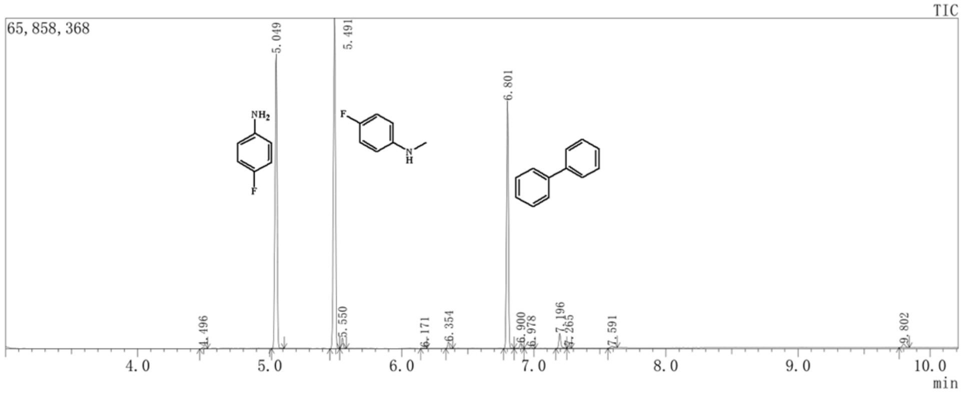

**Figure S19.** GC-MS spectrum of the crude reaction mixture of hydrogenation of 1,3-bis(4-fluorophenyl)urea (Table 2, entry 1) with biphenyl as internal standard.

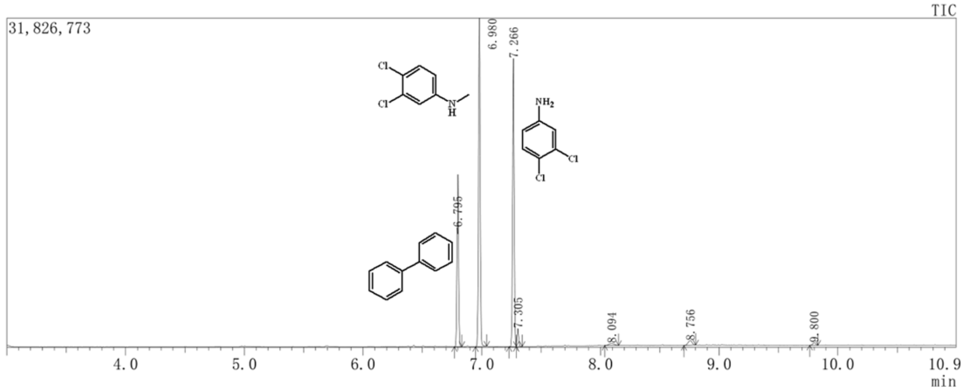


**Figure S20.** GC-MS spectrum of the crude reaction mixture of hydrogenation of 1,3-bis(3,4-dichlorophenyl)urea (Table 2, entry 2) with biphenyl as internal standard.

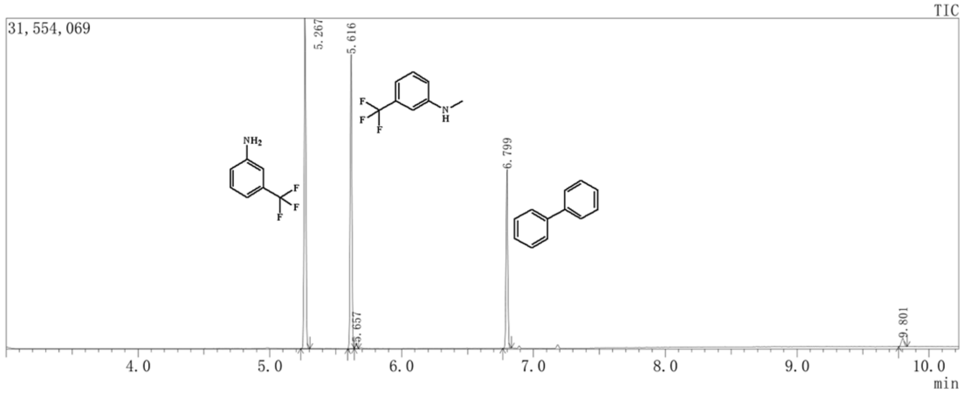


**Figure S21.** GC-MS spectrum of the crude reaction mixture of hydrogenation of 1,3-bis(3-(trifluoromethyl)phenyl)urea (Table 2, entry 3) with biphenyl as internal standard.

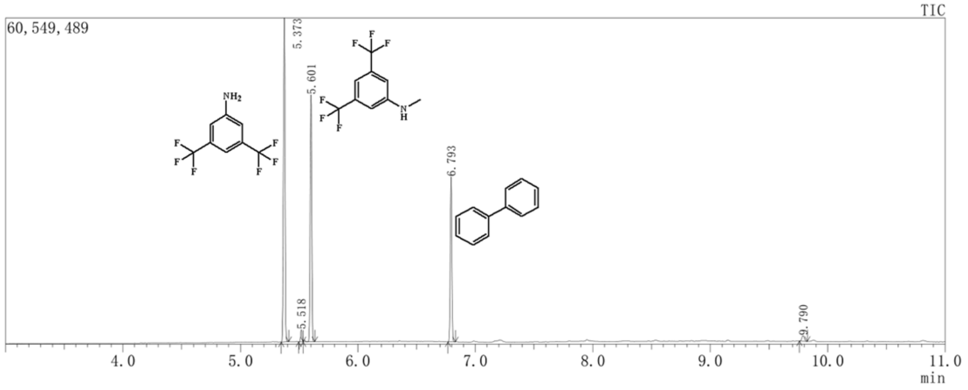


**Figure S22.** GC-MS spectrum of the crude reaction mixture of hydrogenation of 1,3-bis(3,5-bis(trifluoromethyl)phenyl)urea (Table 2, entry 4) with biphenyl as internal standard.

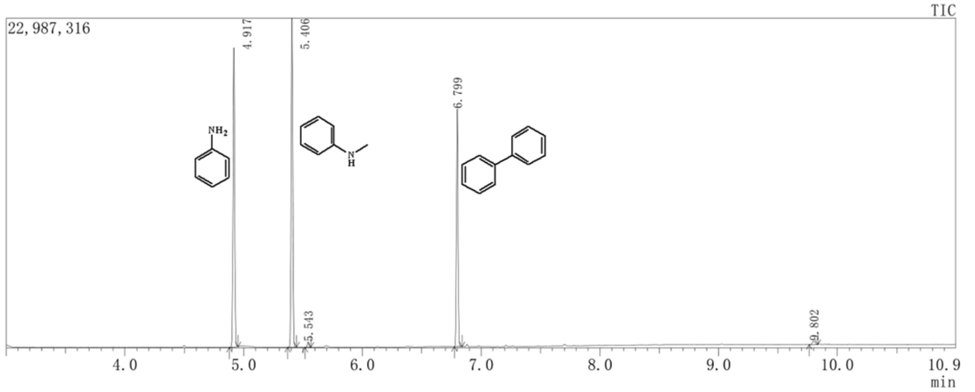


**Figure S23.** GC-MS spectrum of the crude reaction mixture of hydrogenation of 1,3-diphenylurea (Table 2, entry 5) with biphenyl as internal standard.

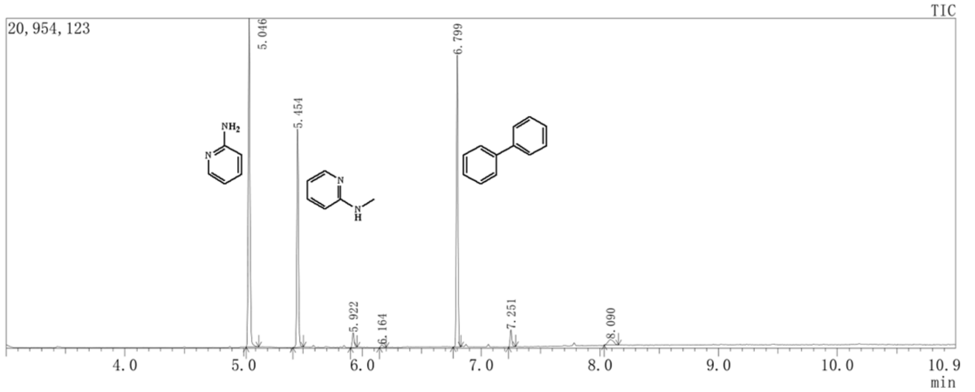


**Figure S24.** GC-MS spectrum of the crude reaction mixture of hydrogenation of 1,3-di(pyridin-2-yl)urea (Table 2, entry 6) with biphenyl as internal standard.

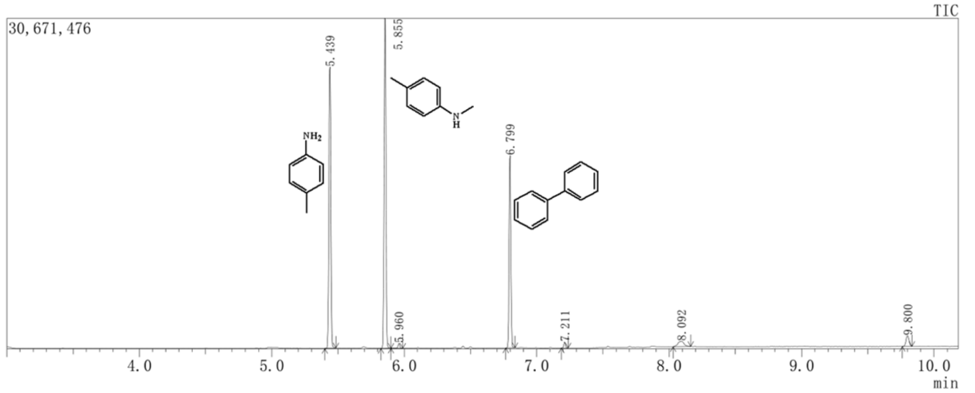


**Figure S25.** GC-MS spectrum of the crude reaction mixture of hydrogenation of 1,3-bis(4-methylphenyl)urea (Table 2, entry 7) with biphenyl as internal standard.

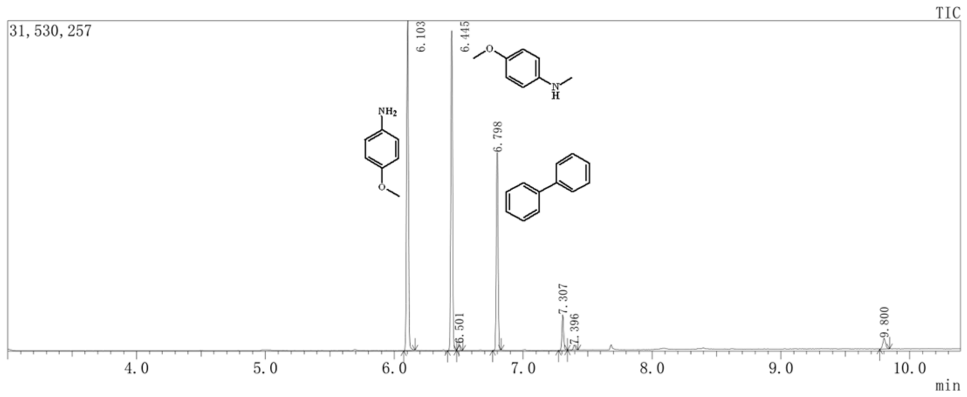


**Figure S26.** GC-MS spectrum of the crude reaction mixture of hydrogenation of 1,3-bis(4-methoxyphenyl)urea (Table 2, entry 8) with biphenyl as internal standard.

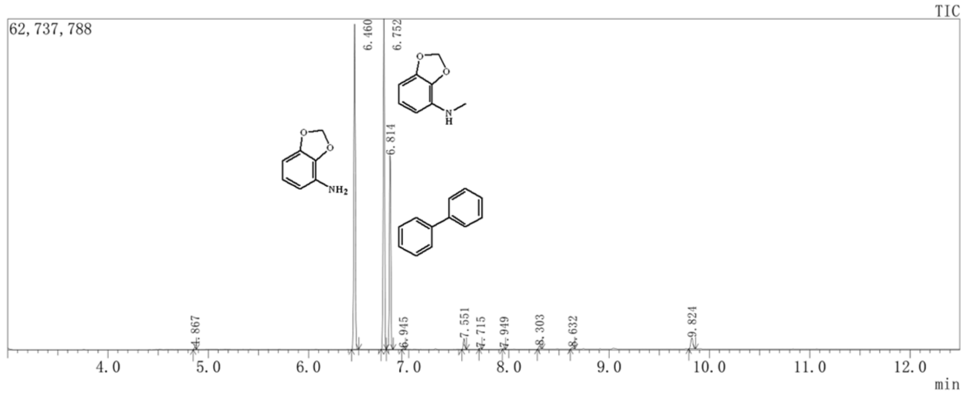


**Figure S27.** GC-MS spectrum of the crude reaction mixture of hydrogenation of 1,3-Bis(benzo[d][1,3]dioxol-4-yl)urea (Table 2, entry 9) with biphenyl as internal standard.

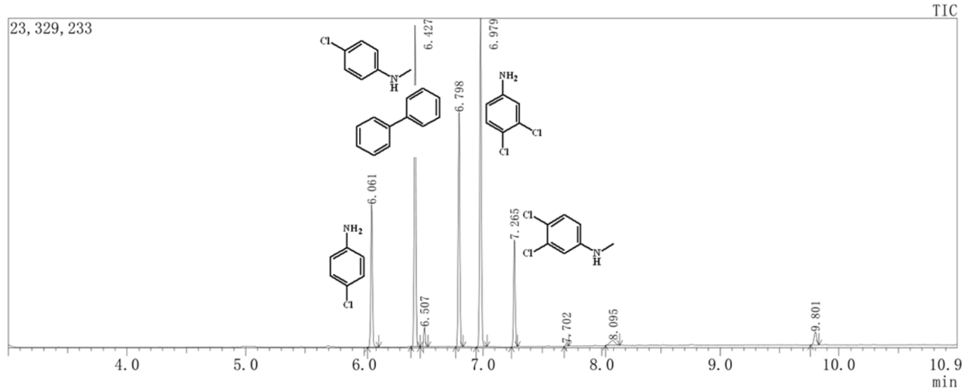


**Figure S28.** GC-MS spectrum of the crude reaction mixture of hydrogenation of 1-(4-Chlorophenyl)-3-(3,4-dichlorophenyl)urea (Table 2, entry 10) with biphenyl as internal standard.

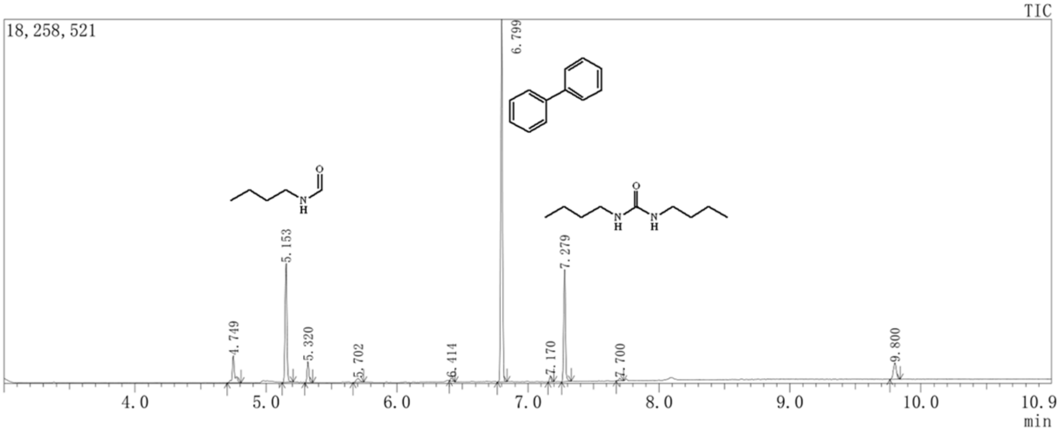


**Figure S29.** GC-MS spectrum of the crude reaction mixture of hydrogenation of 1,3-dibutylurea (Table 2, entry 12) with biphenyl as internal standard.

(b) $^{1}$H NMR Data of Products

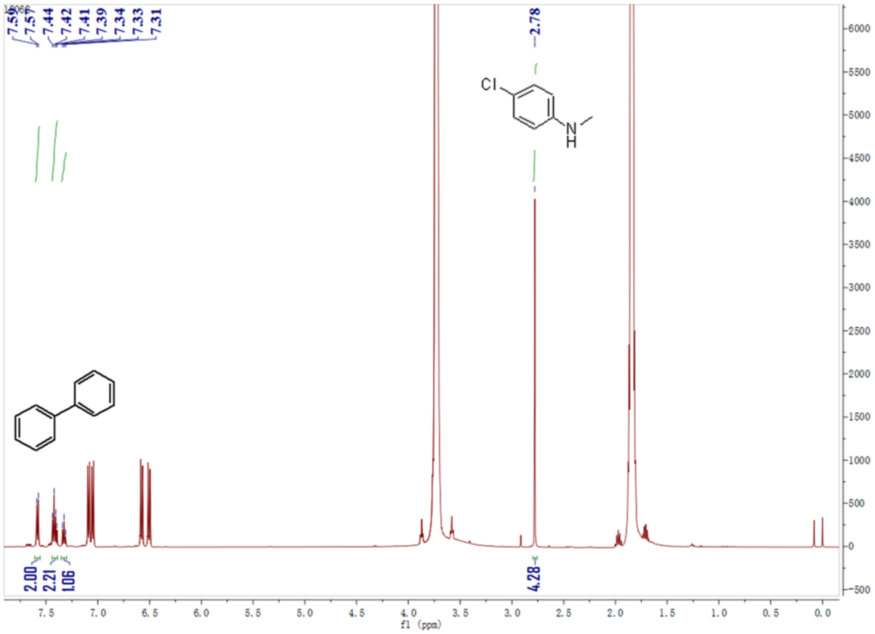

**Figure S30.** $^{1}H$ NMR ($CDCl_3$, 500 MHz) spectrum of the crude reaction mixture of hydrogenation of 1,3-bis(4-chlorophenyl)urea (Table 1, entry 11) with biphenyl as internal standard.

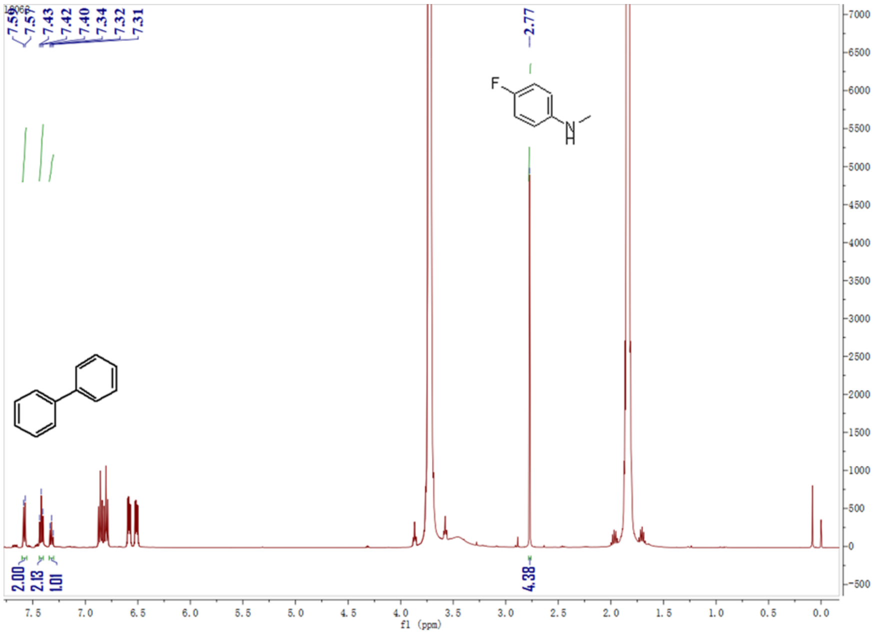

**Figure S31.** $^{1}H$ NMR ($CDCl_3$, 500 MHz) spectrum of the crude reaction mixture of hydrogenation of 1,3-bis(4-fluorophenyl)urea (Table 2, entry 1) with biphenyl as internal standard.

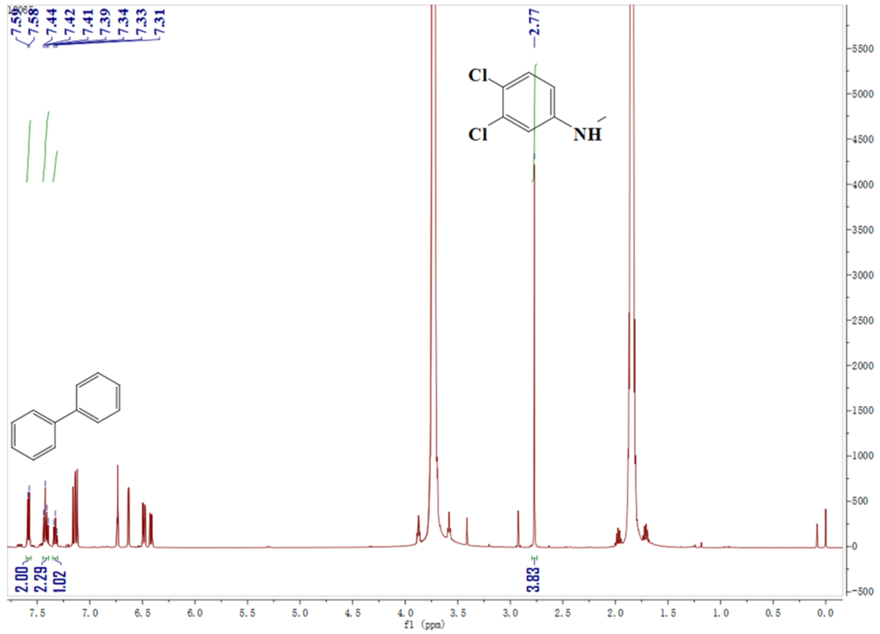


**Figure S32.** $^{1}H$ NMR ($CDCl_3$, 500 MHz) spectrum of the crude reaction mixture of hydrogenation of 1,3-bis(3,4-dichlorophenyl)urea (Table 2, entry 2) with biphenyl as internal standard.

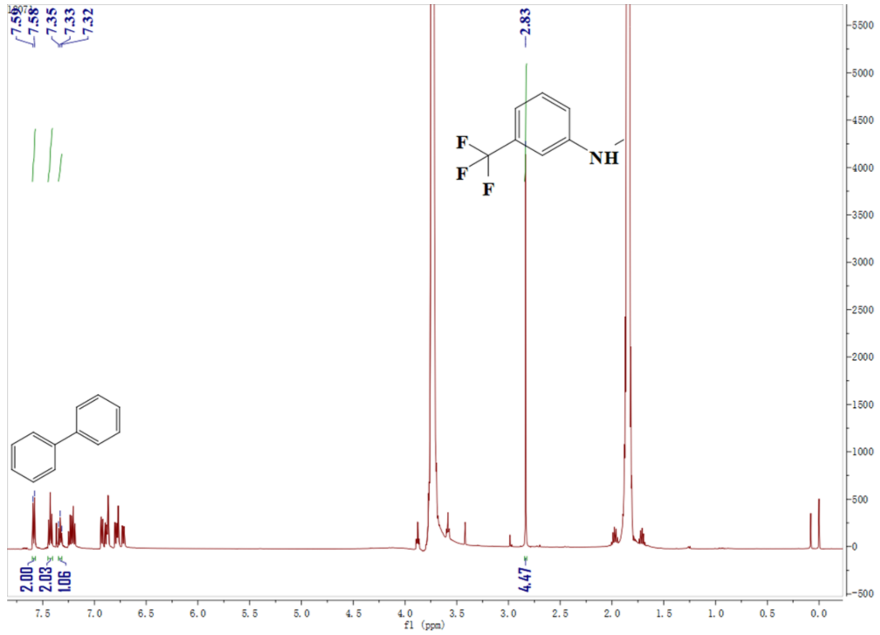


**Figure S33.** $^{1}H$ NMR ($CDCl_3$, 500 MHz) spectrum of the crude reaction mixture of hydrogenation of 1,3-bis(3-(trifluoromethyl)phenyl)urea (Table 2, entry 3) with biphenyl as internal standard.

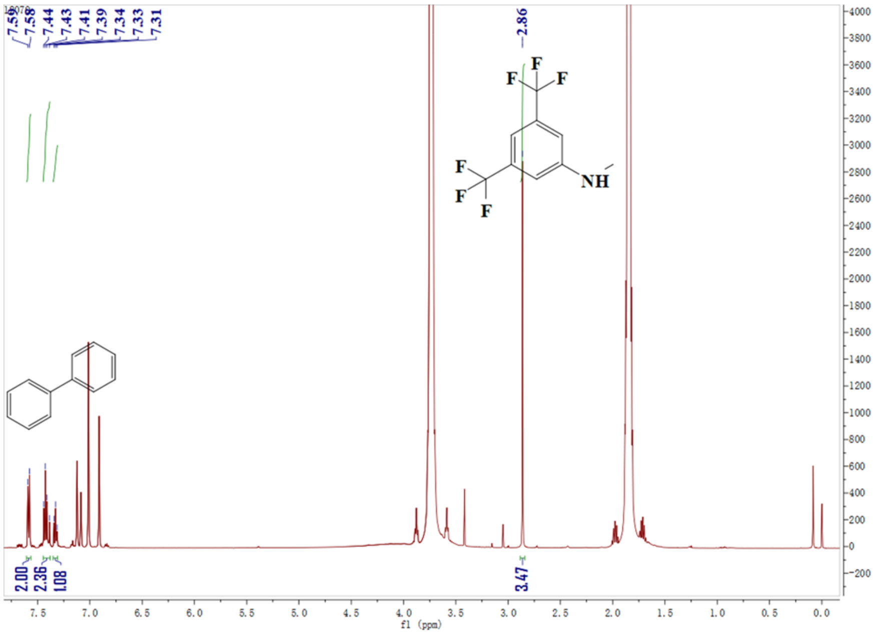

**Figure S34.** $^{1}$H NMR ($CDCl_3$, 500 MHz) spectrum of the crude reaction mixture of hydrogenation of 1,3-bis(3,5-bis(trifluoromethyl)phenyl)urea (Table 2, entry 4) with biphenyl as internal standard.

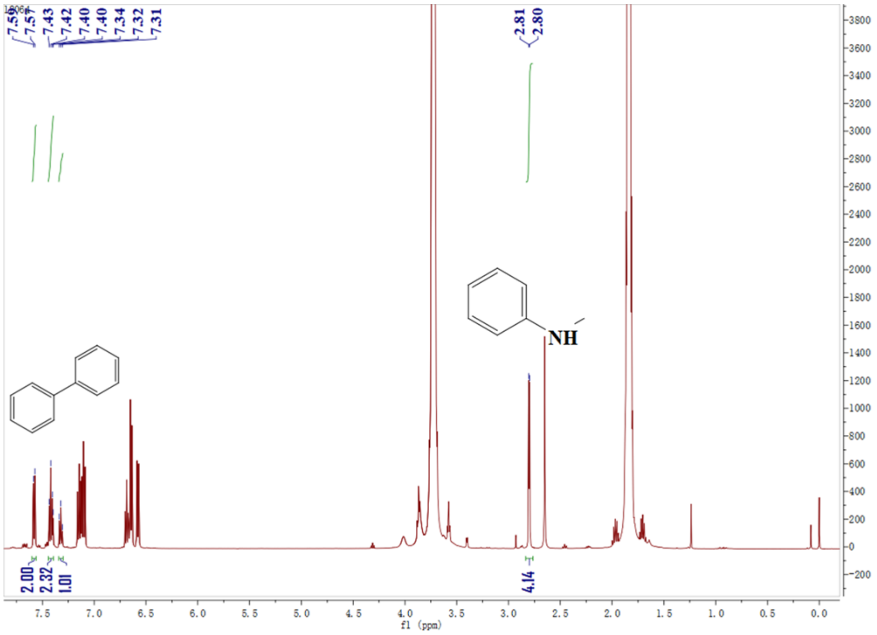

**Figure S35.** $^{1}$H NMR ($CDCl_3$, 500 MHz) spectrum of the crude reaction mixture of hydrogenation of 1,3-diphenylurea (Table 2, entry 5) with biphenyl as internal standard.

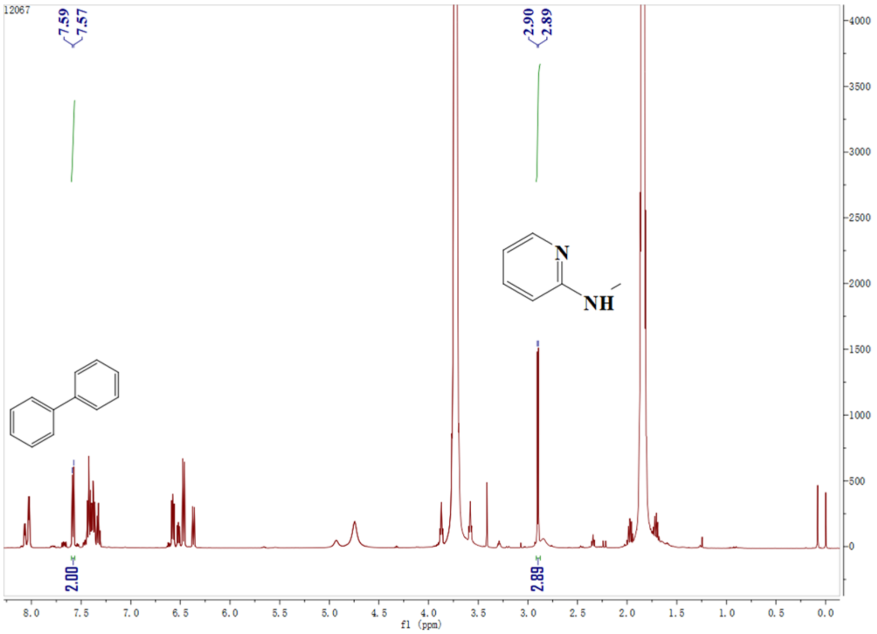


**Figure S36.** $^{1}H$ NMR ($CDCl_3$, 500 MHz) spectrum of the crude reaction mixture of hydrogenation of 1,3-di(pyridin-2-yl)urea (Table 2, entry 6) with biphenyl as internal standard.

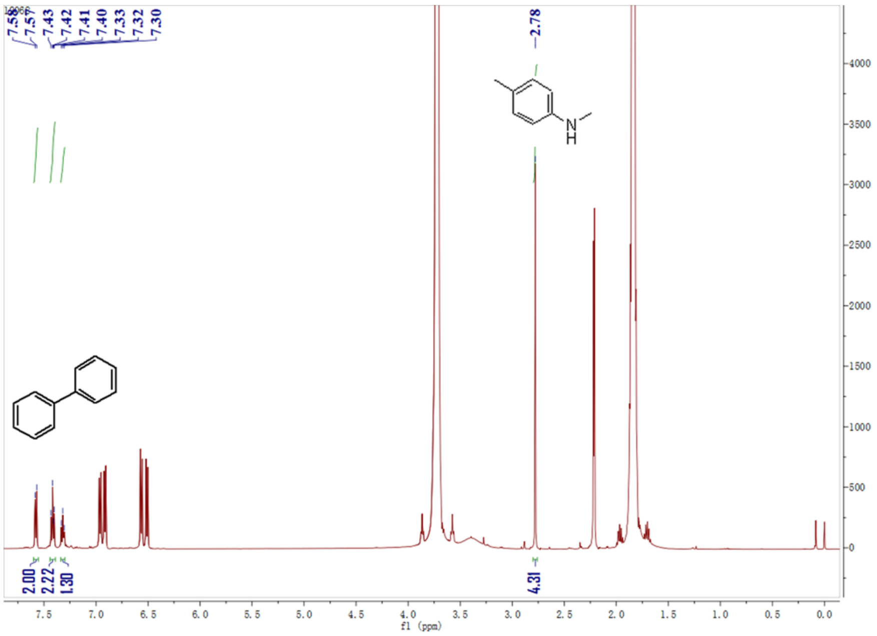


**Figure S37.** $^{1}H$ NMR ($CDCl_3$, 500 MHz) spectrum of the crude reaction mixture of hydrogenation of 1,3-bis(4-methylphenyl)urea (Table 2, entry 7) with biphenyl as internal standard.

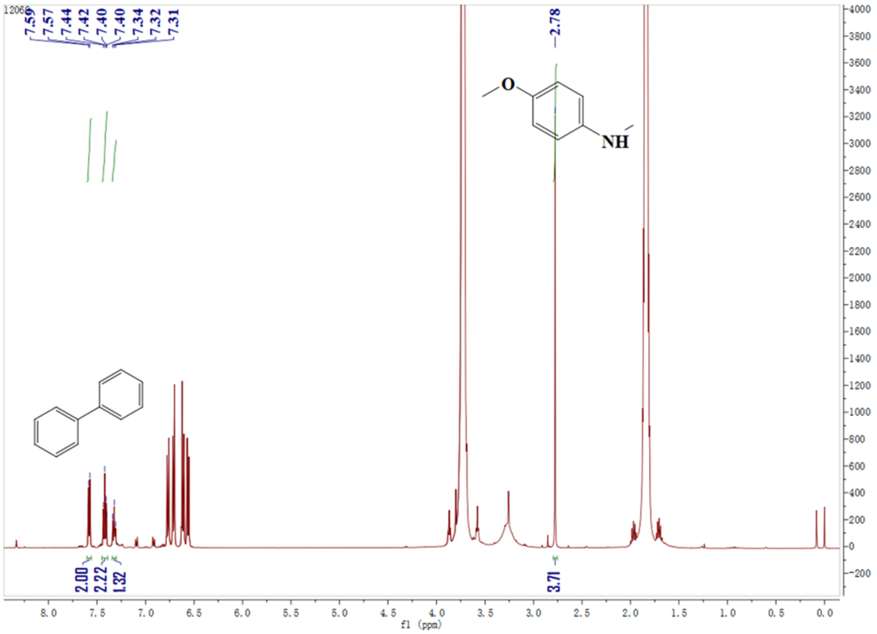

**Figure S38.** $^{1}H$ NMR ($CDCl_3$, 500 MHz) spectrum of the crude reaction mixture of hydrogenation of 1,3-bis(4-methoxyphenyl)urea (Table 2, entry 8) with biphenyl as internal standard.

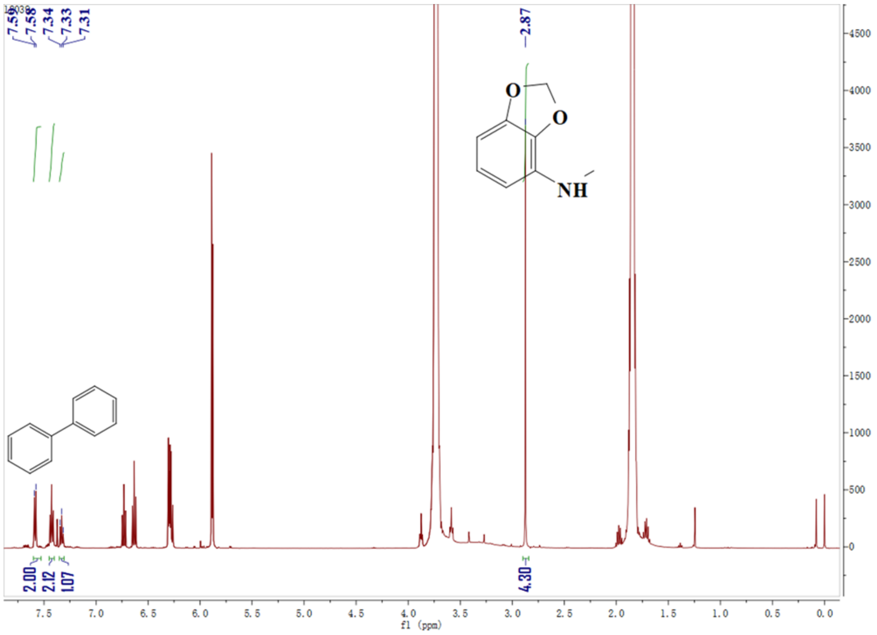

**Figure S39.** $^{1}H$ NMR ($CDCl_3$, 500 MHz) spectrum of the crude reaction mixture of hydrogenation of 1,3-Bis(benzo[d][1,3]dioxol-4-yl)urea (Table 2, entry 9) with biphenyl as internal standard.

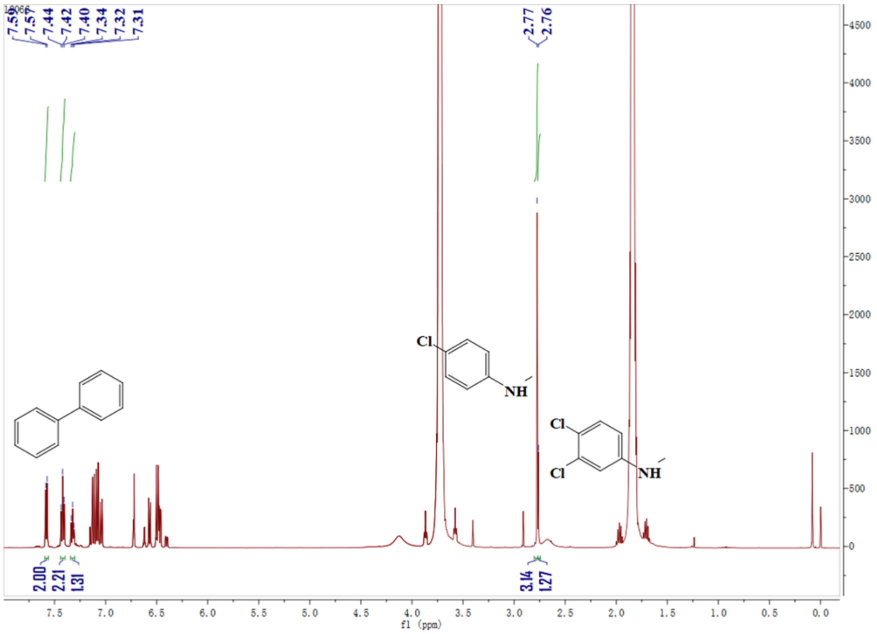

**Figure S40.** $^1$H NMR ($CDCl_3$, 500 MHz) spectrum of the crude reaction mixture of hydrogenation of 1-(4-Chlorophenyl)-3-(3,4-dichlorophenyl)urea (Table 2, entry 10) with biphenyl as internal standard.

(c) NMR Data of *N*-methylaniline Products

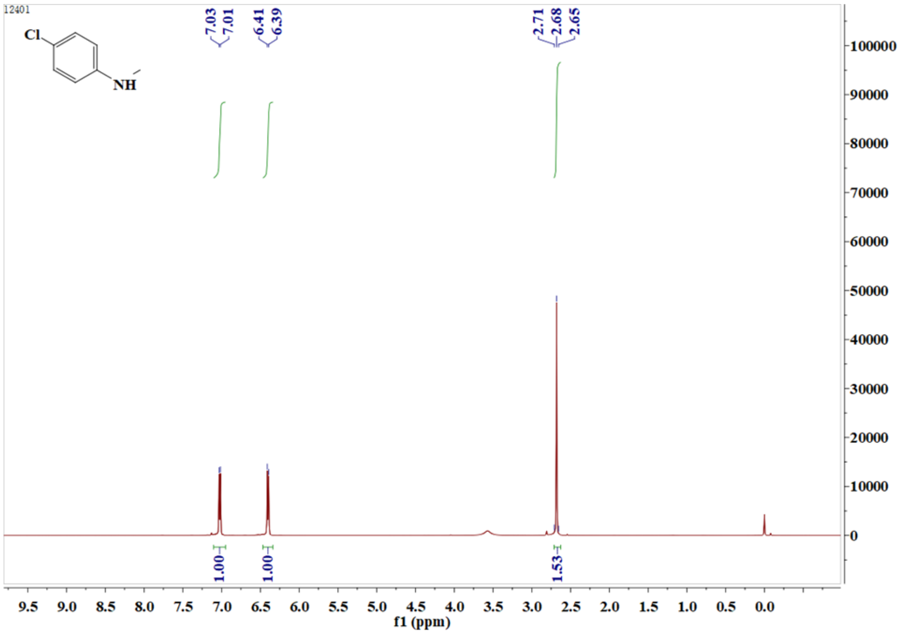


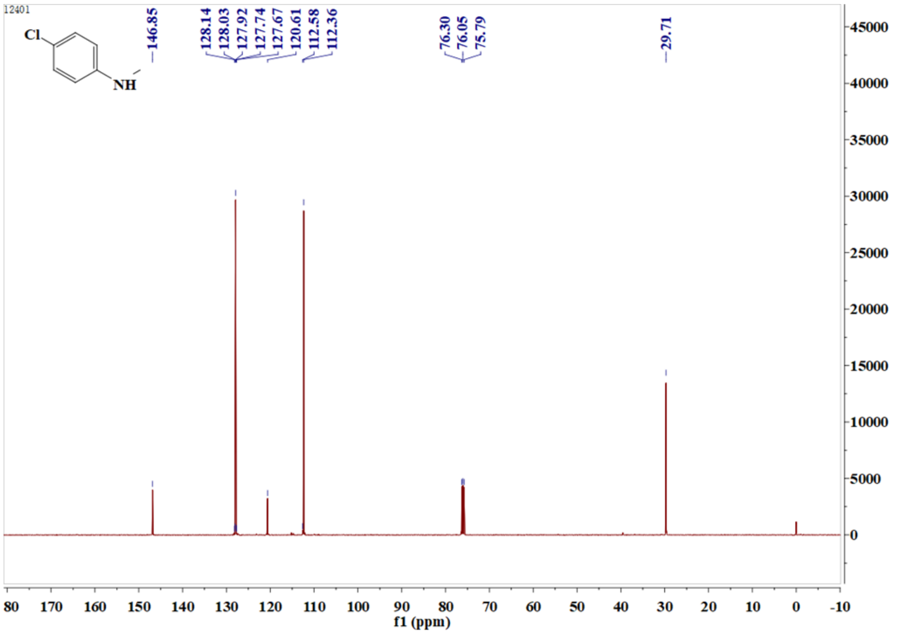


**Figure S41.** $^{1}H$ NMR ($CDCl_3$, 500 MHz) spectrum and $^{13}C$ NMR ($CDCl_3$, 125 MHz) spectrum of the 4-chloro-*N*-methylaniline.

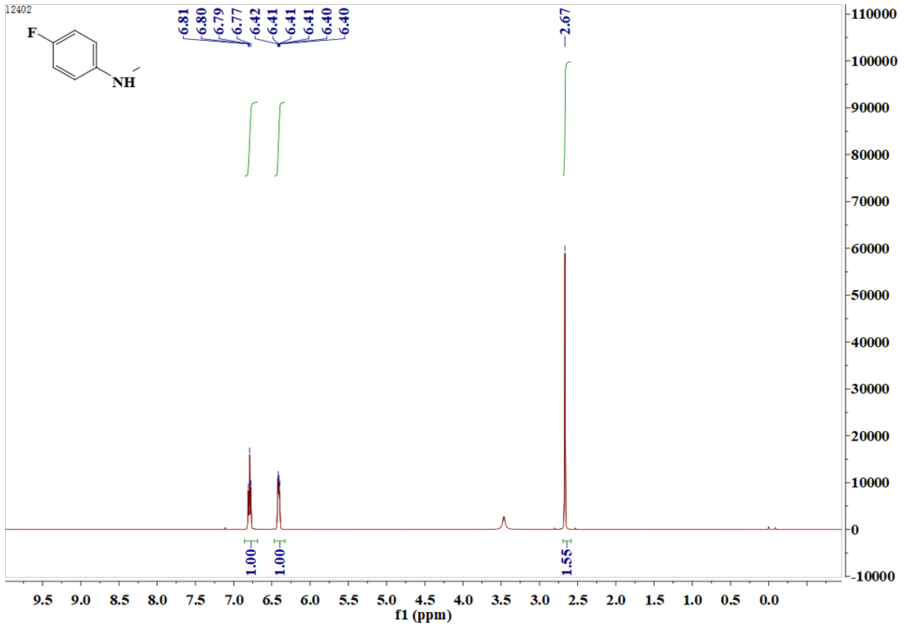

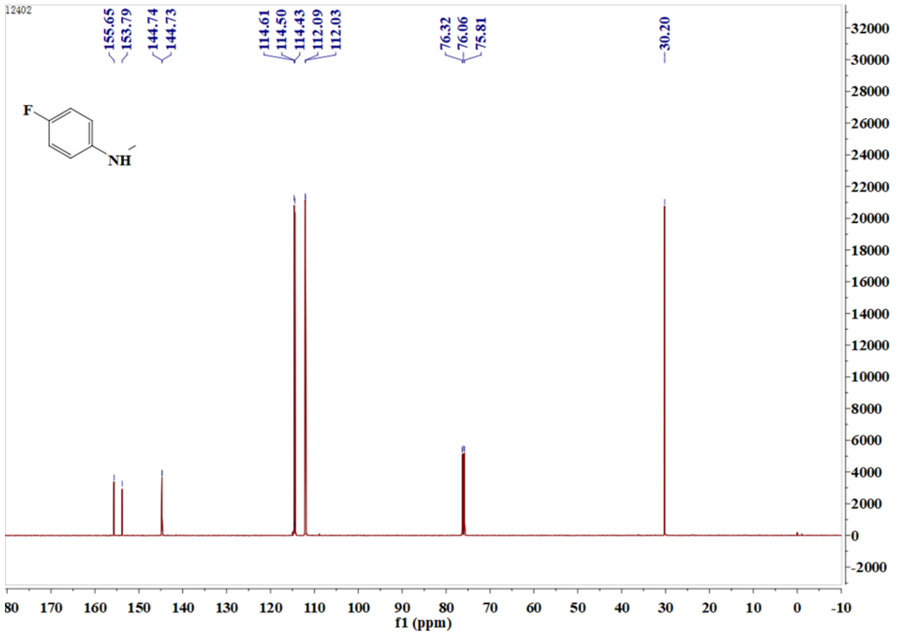

**Figure S42.** $^{1}H$ NMR ($CDCl_3$, 500 MHz) spectrum and $^{13}C$ NMR ($CDCl_3$, 125 MHz) spectrum of the 4-fluoro-*N*-methylaniline.

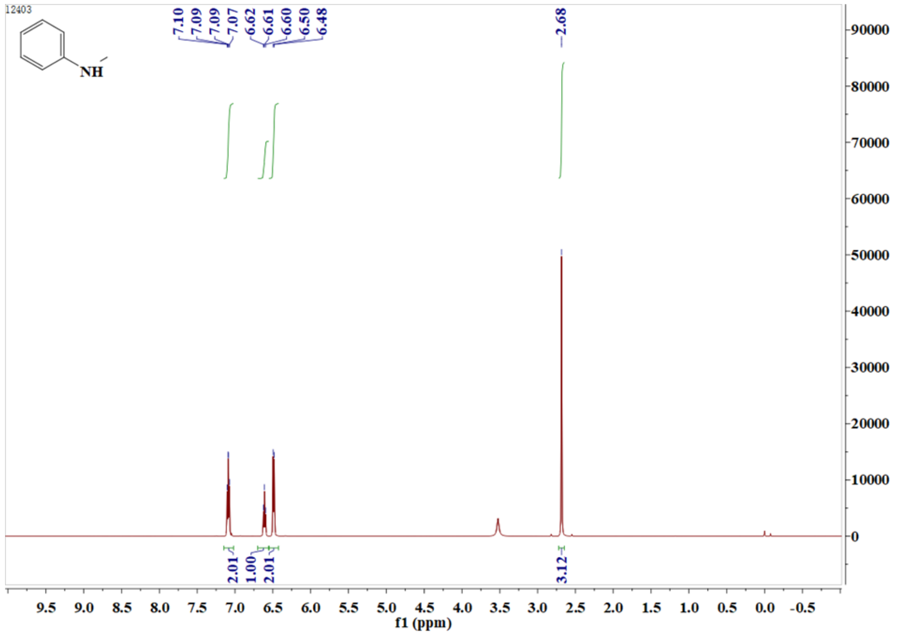


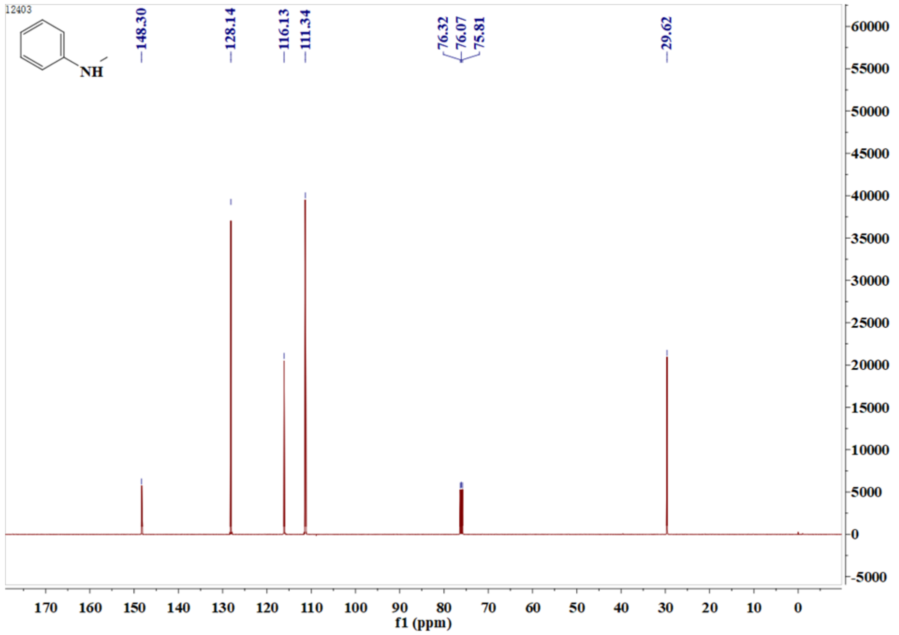


**Figure S43.** $^{1}H$ NMR ($CDCl_3$, 500 MHz) spectrum and $^{13}C$ NMR ($CDCl_3$, 125 MHz) spectrum of the *N*-methylaniline.

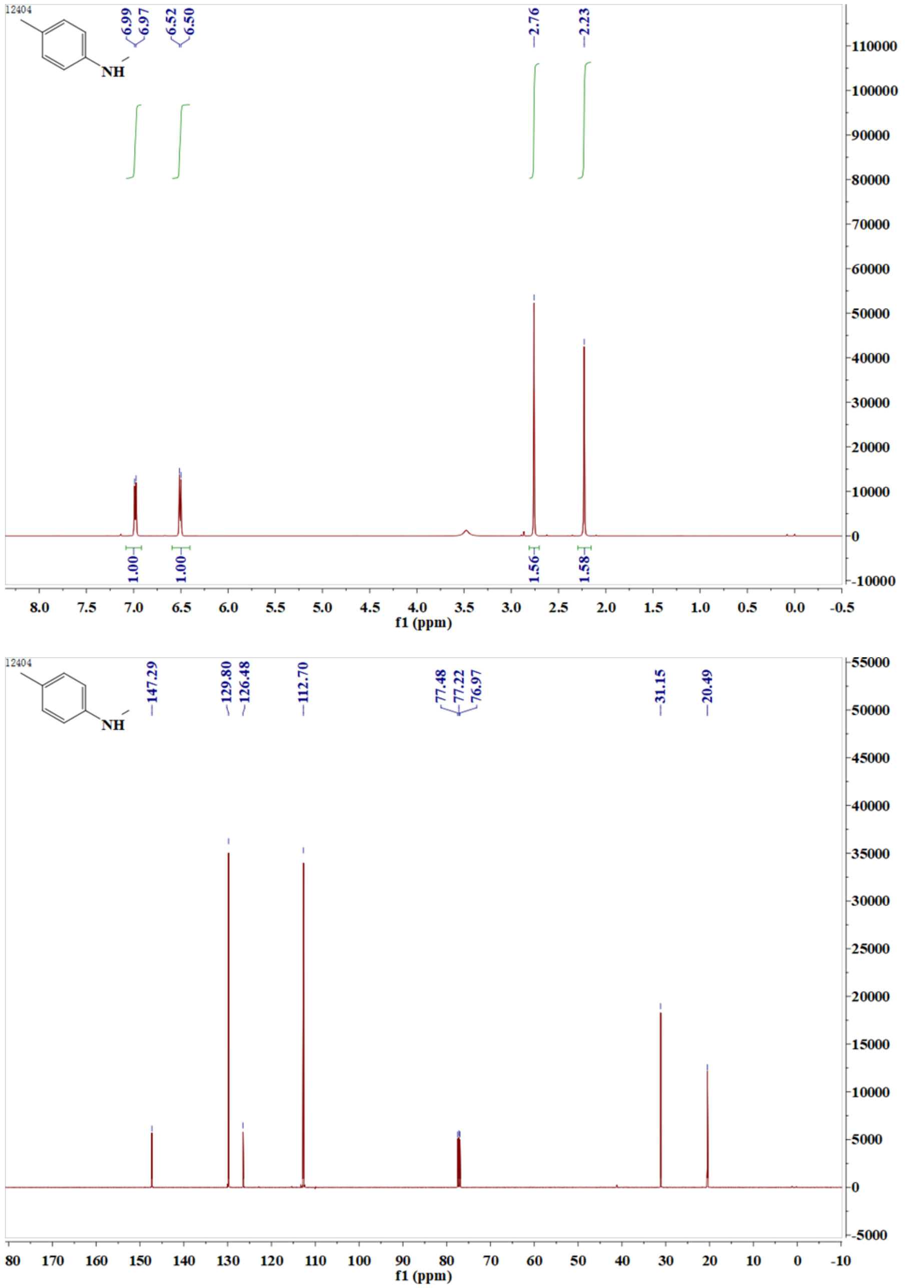


**Figure S44.** $^{1}H$ NMR ($CDCl_3$, 500 MHz) spectrum and $^{13}C$ NMR ($CDCl_3$, 125 MHz) spectrum of the *N,*4-dimethylaniline.

**References**

1. Geilen, F. M.; Engendahl, B.; Harwardt, A.; Marquardt, W.; Klankermayer, J.; Leitner, W., Selective and flexible transformation of biomass-derived platform chemicals by a multifunctional catalytic system. *Angew. Chem., Int. Ed.* **2010,** *49* (32), 5510-5514.

2. vom Stein, T.; Meuresch, M.; Limper, D.; Schmitz, M.; Holscher, M.; Coetzee, J.; Cole-Hamilton, D. J.; Klankermayer, J.; Leitner, W., Highly versatile catalytic hydrogenation of carboxylic and carbonic acid derivatives using a Ru-triphos complex: molecular control over selectivity and substrate scope. *J. Am. Chem. Soc.* **2014,** *136* (38), 13217-13225.

3. Zhu, J.; Zhang, Y.; Wen, Z.; Ma, Q.; Wang, Y.; Yao, J.; Li, H., Highly Efficient Ruthenium-Catalyzed Semihydrogenation of Urea Derivatives to Formamides. *Chem. Eur. J.* **2023**, e202300106.
